# Bridging disciplinary siloes: a scoping review on the inclusion and exclusion of pregnant and lactating populations in clinical research

**DOI:** 10.1136/bmjph-2025-003423

**Published:** 2026-01-14

**Authors:** Mridula Shankar, Alya Hazfiarini, Thiago Melo Santos, Joshua P Vogel, Rosalind McDougall, Annie McDougall, Sara Rushwan, Ahmet Metin Gülmezoglu, Meghan A Bohren

**Affiliations:** 1 Gender and Women’s Health Unit, Nossal Institute for Global Health, Melbourne School of Population and Global Health, The University of Melbourne, Melbourne, Victoria, Australia; 2 Women’s, Children’s and Adolescents’ Health Program, Burnet Institute, Melbourne, Victoria, Australia; 3 Centre for Health Equity, Melbourne School of Population and Global Health, The University of Melbourne, Melbourne, Victoria, Australia; 4 Concept Foundation, Geneva, Switzerland

**Keywords:** Public Health, Scoping Review, Female

## Abstract

**Objectives:**

Despite calls for including pregnant and lactating people in clinical research, exclusionary practices persist. Current arguments for inclusion are fragmented and discipline-specific. This scoping review synthesised how inclusion and exclusion of pregnant and lactating populations in clinical research is framed across fields, and identified recommendations for responsible inclusion.

**Design:**

Scoping review.

**Data sources:**

We searched eight databases from inception to 14 February 2024: MEDLINE, CINAHL, Family & Society Studies Worldwide, SocINDEX, Scopus, Web of Science, Embase and Global Health.

**Eligibility criteria:**

Papers about conceptual or empirical issues related to inclusion or exclusion of pregnant and lactating populations in clinical research. Eligible publications included conceptual analyses, reflections on regulatory and ethical guidance, primary research, review articles, commentaries, viewpoints and editorials.

**Data extraction and synthesis:**

Two reviewers independently screened and extracted data, developed article summaries and iteratively synthesised findings into themes.

**Results:**

We included 188 papers across bioethics, law and regulation, epidemiology, pharmacology and market and product development. We developed 10 themes: (1) narratives of vulnerability, (2) the injustice of exclusion, (3) risk and overprotection, (4) the unique physiology of pregnancy and lactation, (5) business risks in drug development, (6) informed consent and its limitations, (7) evaluating risks and benefits in maternal-fetal therapies, (8) reliance on animal studies for safety data, (9) designing studies for optimal safety and efficacy and (10) the challenges of detecting adverse events. Recommendations highlight early initiation of preclinical studies, consensus on terminology like ‘minimal risk’, standardised trial endpoints, dedicated funding for research networks, incentives for pharmaceutical companies, capacity strengthening for research ethics committees and partnerships with community-based and patient advocacy organisations.

**Conclusion:**

Challenges to the responsible inclusion of pregnant and lactating populations arise across multiple stages of clinical research. Structural changes through coordinated interventions across the research pipeline are needed to change the status quo.

**WHAT IS ALREADY KNOWN ON THIS TOPIC**

- The exclusion of pregnant and lactating populations from clinical research has compromised their health and denied them equitable benefits from biomedical advances.
- Previous analyses of their under-representation have illuminated causes and consequences, yet these analyses have been fragmented, and exclusionary practices remain widespread.

**WHAT THIS STUDY ADDS**

- This study overcomes siloed approaches by mapping literature across bioethics, pharmacology, epidemiology, law and regulation, and market and product development. We synthesised data, consolidating arguments for inclusion and exclusion, along with their critiques and alternative perspectives.
- Our synthesis accounts for the rapidly evolving scope of clinical research, including emerging technologies like maternal-fetal therapies that raise complex, intersecting ethical challenges distinct from those in traditional therapeutic or vaccine research.

**HOW THIS STUDY MIGHT AFFECT RESEARCH, PRACTICE OR POLICY**

- Study findings underscore the importance of aligning research regulations with contemporary ethics and data that supports the responsible inclusion of pregnant and lactating populations in research.
- We emphasise the need for multidisciplinary teams to develop nuanced risk-benefit assessments and adverse event detection. Additionally, creating incentive structures and research funding models that overcome financial disincentives in drug development for these populations is crucial.

## Introduction

 Evidence-based medicine relies on randomised controlled trials to determine if therapies are effective and safe for use.^1^ Human data from trials guide recommendations, clinical decision-making on dosing and mode of administration, and advice on side effects.^2^ Differences in pathways of disease progression and physiological responses to vaccines and medicines require careful consideration of which population groups participate in trials.^3^,^6^ Decades of research and debate have tackled the challenge of inadequate representation of certain participant groups, including pregnant and lactating populations.^7 8^ This under-representation limits the generalisability and impact of clinical research and impedes the provision of evidence-based care to pregnant and lactating women and gender-diverse people.^9 10^ Overcoming some of these barriers, such as protectionist mindsets^11^ and regulatory and market constraints,^12^ has been slow and inconsistent. The scarcity of reliable drug efficacy and safety data during pregnancy and lactation has been labelled a ‘drug drought’,^13^ reflecting the lack of innovations for pregnancy-specific conditions,^14^,^18^ and inadequate evidence to inform vaccine and medication use during pregnancy and lactation for pre-existing conditions.^19^,^22^ Separately, emerging fields—such as in utero surgical interventions and fetal gene therapy—offer new treatment opportunities and introduce unique ethical and regulatory considerations.^23^,^25^

The complex physiological and psychological relationships between the maternal-fetal/baby dyad,^26^ coupled with variable stakeholder perspectives on risks and benefits of research participation,^27^ are important considerations. However, studies have shown that research participation is feasible when pregnant and lactating women have the opportunity to do so, experience therapeutic hope, value the high-quality care provided within trials and hold trust in medicine and research.^27^

While much has been discussed about the exclusion of pregnant and lactating populations from clinical research, we identified two key issues with current argumentation. First, they typically only offer narrow, discipline-specific framings of issues. For example, framing trial exclusion primarily as a bioethics concern,^28^ a pharmacological complexity^29 30^ or a regulatory quagmire.^31^ These siloed definitional arguments are inadequate to fully conceptualise the problem’s scale and have led to fragmented solutions that have not achieved system-wide changes. Second, there have been no attempts to produce a broad, evidence-based overview of the issues and technologies involved. For example, novel technologies to treat fetal congenital conditions in utero are largely experimental and require a weighing of maternal-fetal health interests that are different from, and yet intersect with, challenges to research participation. A 2024 systematic review explored factors (eg, facilitators and barriers) affecting clinical trial participation of pregnant and lactating populations from the perspectives of stakeholders across the research spectrum, as reported in primary research.^27^ However, it did not consider the historical, regulatory, ethical, epidemiological and pharmacological complexities and debates that continue to shape the dynamics of participation, which have been reported in other types of non-primary research (eg, current debates, ethics viewpoints).

To address these gaps, we conducted a scoping review to synthesise how arguments about inclusion and exclusion of pregnant and lactating women and gender-diverse pregnant people in clinical research are framed across fields, critiqued and challenged with alternative perspectives.

## Methods

We report this scoping review using the Preferred Reporting Items for Systematic Reviews and Meta-Analyses extension for scoping reviews^32^ (online supplemental file 1: PRISMA-ScR checklist). The protocol is registered (osf.io/8g9us).

### Topic and population of interest

We included papers that discussed conceptual and empirical issues concerning the inclusion and exclusion of pregnant and lactating populations in clinical research. We considered preclinical studies and clinical trials about therapeutics, vaccines, maternal-fetal therapies (including surgery), and in utero gene therapy. At the outset, we recognise that people capable of pregnancy and lactation have diverse gender identities. We use a range of gender-related terminology to address our focal populations of interest. When we use the terms ‘women’ or ‘maternal’, we do so to (a) align explicitly with empirical or historical source data that is based on experiences of cisgender women or (b) highlight the relationship between social constructions of gender and certain biases that have stymied inclusion of pregnant and lactating populations in research.

We excluded papers that solely reported on barriers and facilitators of participation in, or implementation of, trials with pregnant and lactating populations, as these have been synthesised previously.^27 33^ We excluded papers reporting only on biobanks, fetal tissue research, historical changes to specific human research regulations (eg, the US regulations for protection of human subjects), post-market drug-labelling for pregnancy and lactation, post-marketing surveillance studies, pregnancy registries and database studies. We excluded papers focused only on clinical care concerns among pregnant and lactating populations, rather than research.

### Types of studies

We included conceptual analyses and reflections on practical applications of regulatory and ethics guidance, primary research using quantitative and/or qualitative methodologies, review articles, commentaries, viewpoints and editorials. We categorised papers into fields based on the operational definitions in box 1.

Box 1 **Operational definitions for categorising papers into fields**

**Bioethics:** Papers were categorised as ‘Bioethics’ if they examined ethical aspects of research involving pregnant and lactating populations. This encompassed discussions of bioethical principles, specific ethical frameworks, critiques of existing frameworks and analyses of real-world ethical challenges in conducting (or not conducting) clinical research with these groups.

**Epidemiology:** Papers were categorised as ‘Epidemiology’ if they discussed patterns of maternal and/or fetal conditions and implications for trial feasibility and design, outcome definitions, trial endpoints or diagnostic criteria.

**Law and regulation:** Papers were categorised as ‘Law and regulation’ if they discussed implications of the legal and regulatory landscape on the participation of pregnant and/or lactating populations in clinical research.

**Market and product development:** Papers were categorised as ‘Market and Product Development’ if they discussed financial, liability or other market-related factors influencing development of vaccine and therapeutics for pregnant and lactating populations.

**Pharmacotherapy:** Papers were categorised as ‘Pharmacotherapy’ if they addressed aspects of preclinical research, including animal testing, for assessing pharmacokinetics and pharmacodynamics.

We excluded short communications, interviews, news articles, editorial letters, conference abstracts, workshop summaries, theses, book chapters, book reviews, and regulatory or committee guidance or decisions.

### Search methods

We searched MEDLINE, CINAHL, Family & Society Studies Worldwide, SocINDEX, Scopus, Web of Science, Embase and Global Health, from inception to 14 February 2024. An information specialist developed the search strategy using terms relevant to pregnant and lactating populations, inclusion/exclusion in clinical research, bioethics, pharmaceutics, law and regulation (online supplemental file 2: search strategies). There were no restrictions on publication year, language or geographical setting.

### Study selection

We imported database search results into Covidence.^34^ At least two reviewers independently reviewed titles and abstracts (MS, AH, AM), using Google Translate for records in languages other than those in which the review team were proficient. We retrieved full texts of potentially relevant papers, and two reviewers assessed eligibility independently (MS, AH), with disagreements resolved through discussion with a third reviewer (MAB). Non-English full texts were translated using DeepL translator.^35^

### Data extraction and synthesis

Two reviewers (MS, AH) extracted data on authors, publication year, article type, aims, field (box 1), study setting and research topic (if applicable) and populations of interest (eg, pregnant women, lactating women). Disagreements were reconciled through discussion (MS, AH, MAB). In accordance with methodological guidance for scoping reviews,^36^ we did not conduct quality assessments of included papers. MS and AH developed brief summaries for each article, highlighting specific arguments influencing inclusion and exclusion of pregnant and lactating populations in clinical research, and recommendations to overcome challenges. We synthesised these arguments into broader thematic categories iteratively.

### Patient and public involvement

There was no direct involvement of patients or the public.

## Results

We included 188 papers (online supplemental file 3: PRISMA flowchart). Table 1 summarises characteristics of included papers (online supplemental file 4: characteristics of individual papers). Most addressed issues from the bioethics field (138/188, 73.4%), followed by law and regulation (71/188, 39.4%), epidemiology (51/188, 27.1%), pharmacology (50/188, 26.6%) and market and product development (16/188, 8.5%). Over half (103/188, 54.8%) were classified under two or more fields.

**Table 1:** Summary of characteristics of included papers (n=188)

Characteristics	n	%
Year of publication		
1980–1989	3	1.6
1990–1999	17	9.0
2000–2009	27	14.4
2010–2019	83	44.2
2020–2023	58	30.9
Disciplines*		
Bioethics	138	73.4
Epidemiology	51	27.1
Pharmacology	50	26.6
Law and regulation	71	39.4
Market and product development	16	8.5
Record type		
Case report/opinion/viewpoint	29	15.4
Comment/editorial	42	22.3
Conceptual article	51	27.1
Primary research	3	1.6
Review	53	28.2
Other†	10	5.3
WHO region‡		
Africa	6	3.2
Europe	9	4.8
The Americas	52	27.7
Western Pacific	3	1.6
South-East Asia	2	1.1
Not specified/not applicable	118	62.8
Focal group		
Pregnant person and/or the fetus	157	83.5
Pregnant and lactating persons	31	16.5
Language		
English	183	97.3
French	2	1.1
German	2	1.1
Spanish	1	0.5

* Total percentage exceeds 100% as individual records may be classified under multiple categories.

† Categories included debate, lessons learnt, conference proceedings, methodological article, report, special communication.

‡ Total percentage exceeds 100% as individual records may be classified across more than one WHO region.

Review papers (53/188, 28.2%) were the most common, followed by conceptual articles (51/188, 27.1%). Most papers (118/188, 62.8%) were not specific to a geographic setting; however, the Americas (52/188, 27.7%) was the most common region represented. Pregnant women/people or the fetus were the primary focus in most papers (157/188, 83.5%); 31 papers (16.5%) included lactating women/people.

**Figure 1:**
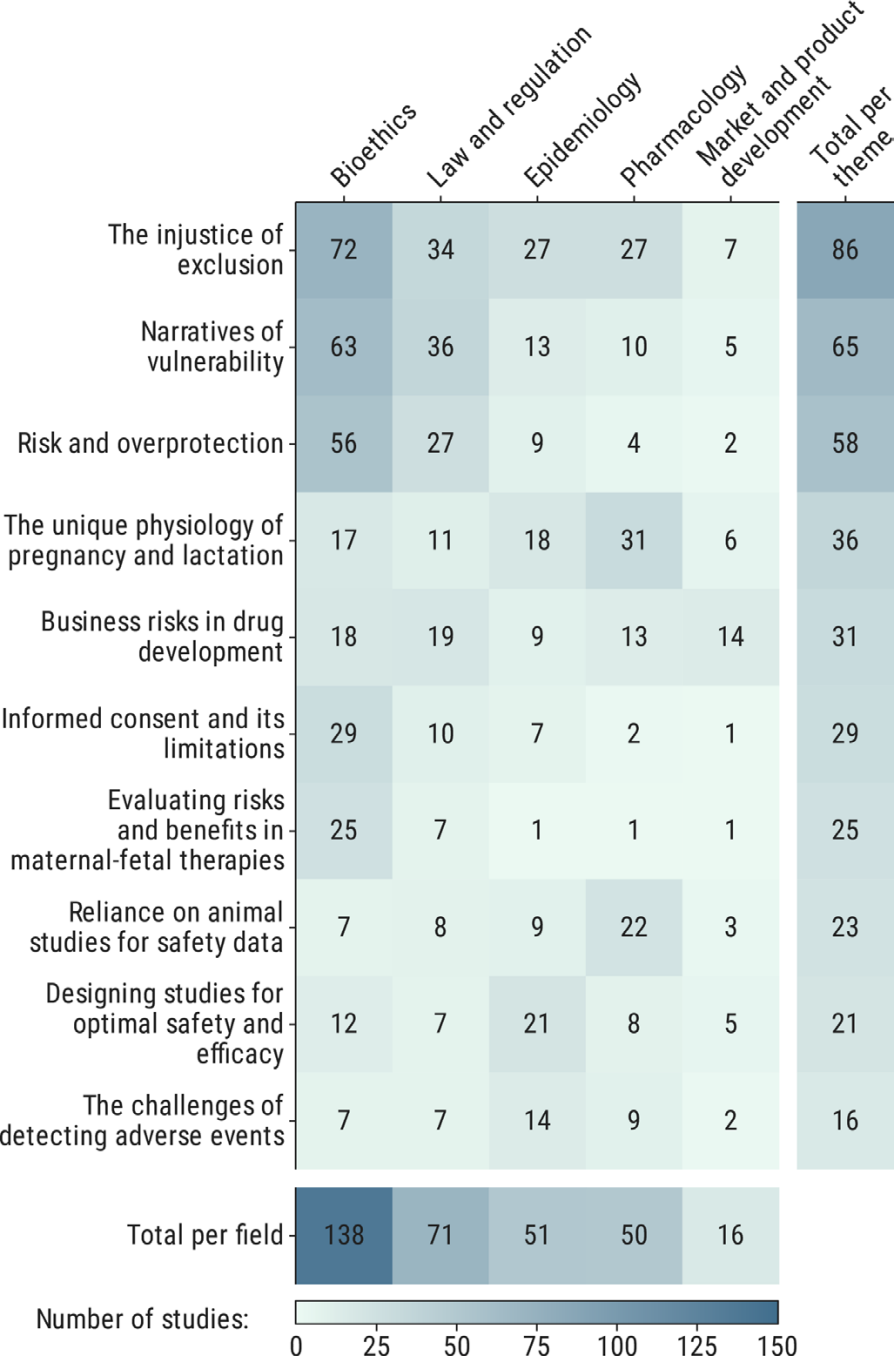
Distribution of papers by themes and fields

We synthesised the data into 10 themes. Figure 1 shows the distribution of papers by these themes and fields. Themes with the highest number of contributory studies were ‘the injustice of exclusion’ (86/188, 50.0%), ‘narratives of vulnerability’ (65/188, 34.6%) and ‘risk and overprotection’ (58/188, 30.9%), with bioethics and law and regulation as the most common fields discussing these themes.

### Narratives of vulnerability

Historical tragedies from use of untested teratogenic drugs (eg, thalidomide)^37^,^44^ and exploitation of disadvantaged populations^42 45 46^ led to regulatory agencies classifying pregnant women as ‘vulnerable’ and in need of special protections.^2837^,^41 45^ In US regulations, ‘vulnerability’ was not formally defined,^45 54 56^ but was central to whether a person could consent to research without coercion.^37 39 56^

Historically, classifying pregnant women as ‘vulnerable’ was justified as protective against potential fetal harm,^3846 55 57^,^63^ yet this signalled diminished decisional capacity during pregnancy.^44 52 60 61 64^ Bioethicists have since argued that assuming impaired cognition during pregnancy is meritless.^3743^,^45 47 56 65^ Even when considering the fetus as lacking decisional capacity, the pregnant woman remains the best person to weigh risks and benefits of research participation.^2344 56 68^,^76^

This model of vulnerability has deterred clinical research in pregnancy, partly due to challenges with obtaining ethics approvals.^46 54 77 78^ This situation has created avoidable harms, including poor outcomes from untreated diseases; prescribing medication without sufficient safety, efficacy and dosing data; and denial of potential benefits from voluntary trial participation.^3739 43 50 56 66 76 79^,^81^ Vulnerability models have also been criticised for failing to distinguish ‘situational’ vulnerability that arises in specific circumstances in pregnancy.^38 43 44 54 58 61 69 76^ For example, obtaining informed consent can be challenging during labour, emergency complications, severe psychiatric illnesses in pregnancy or in the context of experimental treatments for fetal abnormalities.^6063 75 76 78 82^,^87^ In these situations, a woman’s potentially fragile emotional and physical state, the need to make decisions quickly, or the potential for overestimating benefits can complicate the consent process.^60 75 78 83 86 87^

Ethics guidance on vulnerability has evolved over time.^38 88 89^ The Council for International Organisations of Medical Sciences (CIOMS) updated its guidelines in 2016, followed by the 2017 revision (in effect since 2019) of the US Federal Policy for the Protection of Human Subjects (the US Common Rule). Both updates acknowledged that pregnancy does not inherently render an individual vulnerable in research.^3839 51 52 55 66 67 71 90^,^93^ Instead, there has been a shift towards the maternal-fetal dyad being considered ‘scientifically complex’.^3839 51 52 92^,^99^ Despite these changes to minimise exclusion, the persistent perception of vulnerability continues to drive exclusion of pregnant women from research.^67 96^

### The injustice of exclusion

A focus on fetal protection has driven the exclusion of pregnant populations from vaccine and therapeutic trials.^2628 44 49 51 52 59 62 67 70 74 82 92 96 99^,^108^ This precautionary approach overlooks the importance of the bioethical principle of justice, which demands fair distribution of the risks and benefits of research^2628 38 44 46 49 50 52 56 61 64 70 74 81 91 92 94^,^96 98^ and, when necessary, corrective action to address past exclusion.^107 114^ Specific concerns regarding justice in this context gained prominence in the 1990s when HIV-positive pregnant women were excluded from most clinical trials, except those on perinatal transmission, which subsequently brought significant health benefits to the maternal-fetal dyad.^44 46 55 61 92 96 97 99 104 105 115 122 124 125^ The injustice of pregnancy and lactation-related exclusion has persisted into contemporary HIV, Hepatitis C, Zika, Ebola and COVID-19 trials.^5055 59 65 67 68 71 81 90 92 94^,^97 101 109 113 114 117 122 126^

An exclusionary approach creates injustice at both individual and population levels. At an individual level, it prevents pregnant and lactating people from accessing trial benefits, including high-quality care and promising experimental therapies.^26 56 68 74 81 94 96 105 109 111 113 115 116 119^ At a population level, it stymies access to new interventions and prolongs reliance on poorly studied treatments in clinical practice.^2638 56 65 67 68 74 81 89 92^,^94 96 99^ Pregnant persons sometimes need treatment for pre-existing conditions,^82 91 131 133^ novel therapies for obstetric complications^38 80 110 111^ or lifesaving interventions during health emergencies like Ebola or COVID-19.^65 81 114 117^ However, exclusionary practices mean there are limited data on the safety and efficacy of medicines and vaccines during pregnancy.^74 90 92 93 95 107 108 111 117 118^ This limited data forces clinicians and patients to make decisions ‘in the dark’ about their benefits and side effects.^728 41 49 51 56 62 74 90 92 97 100 109^,^111 114 116 118 128 134^ Many opt to discontinue treatment, refuse medication or take medications at ineffective or excessive doses without proper monitoring—collectively, this threatens the health of the maternal-fetal/baby dyad.^2838 49 52 55 65 70 74 82 89 91^,^93 95 98^

Some advocate for a paradigm shift to presumptive inclusion of pregnant populations.^42 55 59 74 89 95 100 101 110 116 142^ Others acknowledge that presumptive inclusion is aspirational but challenging to achieve without (1) substantial changes to preclinical research, enabling clearer understandings of whether and how interventions may work differently in pregnancy^107 133^ and (2) buy-in from diverse stakeholders, including funders, regulators, ethics committees, researchers and potential participants.^7 29 51 52 54 65 76 89 94 104 107 108 121^

### Risk and overprotection

Regulatory guidance on inclusion of pregnant women in research has been criticised for protectionist approaches^48 55 72 104 143 144^ and ambiguous language, particularly in assessing risk across pregnancy stages.^2831 39 53 60 88 89 96 123 138 145^,^147^ For instance, the US Common Rule specifies that for research with no prospect of benefit (eg, Phase 1 trials), the risk to the fetus should be minimal.^3996 147^,^149^ Minimal risk is defined as ‘the probability and magnitude of harm or discomfort anticipated in the research are not greater than those typically encountered in daily life or during routine physical or psychological examinations’.^39 96 97 147^ Applying this definition to the fetus is not straightforward.^147149^,^152^ One approach is to apply an absolute standard—equating minimal risk to the fetus with risks typically experienced by healthy pregnant women in daily life or routine care.^93 147^ However, perceptions and experiences of daily risk vary based on social, economic, environmental and geographical contexts, gestational age and degree of ill-health.^39 93 96 97 147^ An overly cautious interpretation of this definition as ‘no risk’, rather than reasoned and calculated risk, results in exclusion.^48 74^

An excessively restrictive stance can create an adversarial dynamic, positioning the fetus as requiring greater protection than the pregnant woman, or protection from the woman herself.^48 72 79 122 125 153^ This can foster maternal-fetal conflict,^38 46 55 72 74 96 124 139^ essentially excluding women from decision-making and violating their decisional autonomy.^46 55 89 95 96 114 122^ Such conflict arises when there are risk-benefit trade-offs between the woman and her fetus.^55 74 79 86 139 154^ Resistance to any increase in potential fetal risk from unknown adverse effects of an experimental treatment—regardless of potential benefits to the woman—reinforces this conflict.^48 74 125^ However, if an experimental treatment only poses risks to the woman, these risks can be justified if there are potential benefits to the fetus. Thus, a double standard is created: fetal risks are grave, while maternal risks are acceptable.^48 155^

Chervenak and McCullough introduced the ‘fetus as a patient’ concept to underpin ethical criteria for initiating clinical studies in pregnancy.^85 112 156 157^ First developed in the context of fetal surgery,^112 156 157^ and later expanded to other interventions,^158^ this concept emphasises that a viable fetus (ie, one that can live outside the uterus) possesses a ‘dependent moral status’, and that clinicians have beneficence-based obligations to protect its life, health and interests as a person in the future.^85 112 157 158^ These obligations must be balanced with those to the pregnant woman, including beneficence and respect for her autonomy.^85 112 157 158^ The authors offer guidance on when it is ethically appropriate to (a) initiate Phase 1 and 2 trials and (b) advance to Phase 3 trials in pregnancy.^158^ They state that early-phase fetal research needs to demonstrate that (1) the intervention is reliably expected to be life-saving or prevent injury or disability, (2) poses least risk of fetal morbidity or mortality and (3) has low risk of maternal morbidity or mortality.^112156^,^158^ Chervenak *et al*’s framework has been applied in ethical analyses, including support of the existing US regulatory framework,^148^ placental therapies,^159^ inclusion of pregnant women with HIV or hepatitis infections in maternal-fetal therapy trials,^23^ and justifying placebo-controlled trials for mental health conditions during pregnancy.^131 160^

However, the concept of fetal patienthood has been criticised by bioethicists.^26142 146 154 161^,^165^ Criticisms include the lack of definitional clarity on key terms, such as the meaning of ‘reliably’ in relation to the expectation that the intervention will confer benefits,^161^ and the definition of fetal ‘dependent moral status’.^165^ Others argue that the ‘intervention poses least risk of fetal mortality’ criterion is too stringent, as most fetal procedures carry a greater than minimal risk.^164^ Additionally, the framework does not address situations where clinicians may decide to end a pregnancy early for a pregnant woman’s health or survival (eg, pre-eclampsia/eclampsia), thus risking fetal health.^164^ The concept of ‘fetus as patient’ also blurs distinctions between patients and participants to whom clinicians and researchers owe distinct and different obligations.^26 154 166^ It oversimplifies the inter-connectedness of the maternal-fetal dyad,^26 43 162 163^ encourages beliefs that obligations to pregnant women and the fetus are equal,^26 164^ and may lead to therapeutic misconception in early phase trials where there is no prospect of direct benefit to the participant.^26^ By its design, the framework also removes decision-making power regarding research participation from the pregnant woman to the attending clinician.^142^

Separately, Chervenak and others argue that abortion preferences cannot ethically be used as a basis for enrolment,^85 157 167 168^ though this stance is contested particularly for maternal-fetal therapies to address fetal conditions.^169^ Since the purpose of such research is to evaluate fetal and infant outcomes post intervention, investigators may choose to recruit women who carry their pregnancies to term.^169^ However, this preference could pressure participants to maintain pregnancies, compromising reproductive autonomy.^170^ In jurisdictions with abortion restrictions, women may feel compelled to participate in a maternal-fetal intervention due to constrained choice.^170 171^

### The unique physiology of pregnancy and lactation

Physiological changes during and after pregnancy can alter a products’ pharmacokinetics, including absorption, distribution, metabolism and excretion—this can affect safety and efficacy of drugs in pregnant or lactating women.^920 29 50 51 60 62 74 82 89 95 100^,^102 106 110 118 120 122 128 134 136 138 172^ Conducting trials across gestational ages allows the identification of ‘critical periods’ of risk of exposure for the fetus.^120^ The fetus can be exposed to an experimental product if it can cross the placenta,^51 136 173 176 178^ though this is not necessarily harmful.^94^ The placenta has selective mechanisms that can limit placental transfer.^176 178^ Thus, fetal exposure depends on factors like the amount of drugs or active metabolites crossing the placenta,^178^ dose-response in the fetus at different gestational ages,^173 178^ placental macroscopic and microscopic features,^173^ and placental drug seizure.^173^

After pregnancy, further drug dosing adjustments may be needed given changes to a woman’s postpartum physiology.^29 179^ The physiological states of pregnant and lactating women are distinct, and they should not be grouped together as a single population.^67 102 138 179 180^ Differentiating pregnant and lactating women can also help identify different pathways to a baby’s exposure. Babies might be exposed to pharmaceutical products through breast milk, though this depends on physiochemical characteristics such as plasma protein binding affinity, degree of ionisation and molecular size.^136 176^ The excretion of pharmaceutical products in breast milk does not necessarily correlate with toxicity, such as vaccine-induced maternal antibodies through breast milk.^94 136 176 180 181^

### Business risks in drug development

Financial considerations are a major limiting factor for pharmaceutical companies to pursue research and development for products used during pregnancy and lactation.^60 140^ Companies may perceive the market for such products as too small and financially risky, given the transient nature of pregnancy, declining fertility rates and the comparatively short duration of most obstetric complications.^159 178 182 183^ Additionally, obtaining accurate data requires large sample sizes and high insurance liabilities,^29 31 89 140 174 178 182 184^ leading to high trial costs.^178 179^ Companies are concerned at the unquantified risks of teratogenicity,^51 59 125 151 182^ exacerbated by the limited preclinical, efficacy and safety data available.^29^ Companies are unlikely to invest in research to evaluate any teratogenic risks as this is expensive, time-consuming and may delay drug release, impacting sales.^89 121 134 182^ This contributes to fears of costly liability and litigation.^29 31 49 50 76 85 89 91 103 115 121 140 148 174 178 182 184^ For example, lifelong settlement costs for infants harmed in utero can reach £5 million in the UK. In the USA, the jury-driven tort system often favours punitive damages, reaching as much as US$110 million.^182^ Despite laws and regulations addressing liability and litigation costs, wide variation across regulatory bodies has led to unclear directives on including pregnant women in trials.^39 185^ Consequently, excluding pregnant women altogether can be seen as a reasonable approach to mitigate liability risks.^53 151^

Enforcing pharmaceutical companies’ accountability is challenging. To establish liability, a causal link must be demonstrated between the breach of care and the harm incurred,^4 151 184^ but gaps in safety and efficacy data make it difficult to establish causation.^4 151^ In English and European tort law, the fetus is not considered a legal entity until birth, so claims are usually filed for injuries to the woman rather than the fetus.^151^ Although some regulations recommend voluntary compensation for research participants, they lack binding provisions for fetal injuries, highlighting gaps in fetal injury protection in clinical trials.^151^

Stringent drug safety regulations for pregnancy, ethical and quality standards for conducting human research, and the limited number of drugs in the pipeline have heightened the risk of revenue losses, making companies increasingly risk-averse.^31 182 186^ In this environment, off-label drug use for pregnant and lactating women is commonplace, as it bypasses the need for costly safety and efficacy data.^82 89 182 183^

### Informed consent and its limitations

Informed consent is a procedural and legal mechanism that aims to respect individuals’ autonomy to decide voluntarily whether to participate in research.^79 84 93 144 153 187 188^ There is consensus that pregnancy does not automatically impair a person’s ability to assess the risks and benefits of research to the maternal-fetal dyad, or to make reasoned decisions about participation.^28 44 46 48 52 64 74 79 105 144 153^ Yet the process of information-sharing and decision-making is not purely procedural.^84 139 148^ It requires that potential participants are fully informed, feel culturally safe, are free from pressure and are able to deliberate.

Transferring informed consent procedures across culturally, socioeconomically and linguistically diverse regions presents distinct ethical challenges.^86150 187 189^,^192^ These include adapting consent procedures to ensure cultural congruence and meaning, while adhering to international ethical standards. Consent involves cultural competence of how gender roles influence decision-making, ensuring voluntariness and understanding, and navigating how countries and institutions differ in their conceptualisation of ethical standards.^86150 187 189^,^192^

Guidelines and regulations on paternal consent are not uniform.^193^ For instance, CIOMS guidelines suggest that pregnant women may choose to consult with the father, whereas the US Common Rule requires (aside from exceptional circumstances) maternal and paternal consent when the intervention potentially benefits the fetus directly.^39 46 70 73 84 122 144 148 193^ Ethical analyses examining paternal consent raise issues such as the father’s interests in fetal and neonatal well-being, the potential impact of trial outcomes on the father’s quality of life, the gathering of biological and genetic information that could affect a father’s privacy and the role of the father as a legal representative on birth.^122 159^ These considerations must be weighed against the potential for paternal consent to infringe on women’s autonomy^58 69 122 144 153 193^ and right to confidentiality,^144^ and its lack of recognition of sex and gender minority family formations.^122^

### Evaluating risks and benefits in maternal-fetal therapies

Advances in prenatal diagnostics to identify congenital anomalies have been accompanied by a growth in prenatal therapies such as surgery,^23 25^ medicines,^194^ fetal gene therapy^167 168^ and reproductive genomics.^24^ Many are experimental due to uncertain efficacy evidence,^166 195^ yet they have evolved as innovations^157 196 197^ without robust clinical trial evidence^25 195^ (eg, fetal endoscopic tracheal occlusion for congenital diaphragmatic hernia). When innovative treatments are available clinically, it is difficult to argue clinical equipoise,^163^ and women may avoid trial participation if they believe they will miss out on therapeutic benefit.^25 163^

Maternal-fetal surgery is further complicated by the pregnant woman bearing the treatment-related risks, and any benefits solely for the fetus.^24 195 197^ While informed consent is appropriate for individual decision-making, the unique maternal-fetal relationship, coupled with societal expectations of motherhood, raises concerns about this model. Concerns include the potential for therapeutic misconception^63 157^ and whether women feel compelled to behave altruistically for fetal health,^25 44 63 73 75 84 157 159 196^ conferring ‘deferential vulnerability’ to the pregnant person.^69^ The ethical challenges are greater in multiple pregnancies, where addressing an anomaly in one fetus may expose the other fetus to risk.^198^

Regulatory guidance has long been insufficient for nuanced risk-benefit analyses on maternal-fetal interventions.^28 73 149 171 195 199^ In 2022, Hendriks and colleagues introduced an ethical framework for these situations, aiming to overcome the historical reliance on categorical thresholds for risk-benefit assessments (eg, ‘only in life-threatening conditions’), and the notion that benefits can only be biomedical in nature.^73^ They argued that categorical thresholds invalidate a more nuanced risk-benefit analysis, particularly for novel technologies—thus hindering innovation. Additionally, narrowly considering biomedical benefits overlooks broader psychosocial benefits to the woman and baby. For example, the authors argue that when considering fetal myelomeningocele repair for spina bifida, risk-benefit considerations should also include improvements in quality of life, emotional well-being and self-efficacy. A study’s overall risk-benefit ratio must therefore include its social value, alongside assessments of the risks and benefits for the pregnant woman and the fetus separately. They emphasise assessment of psychosocial benefits, and recommend appropriateness to proceed if the risk-benefit ratio of the study overall is favourable, even if the risk-benefit ratio is slightly unfavourable to the woman. The main criticisms of this framework are its failure to consider reproductive choice, as it assumes that individuals considering an intervention want to continue the pregnancy, a concern that is heightened when abortion is restricted.^24 170 171^

### Reliance on animal studies for safety data

Data on the safety and risks of pharmaceutical products in pregnant and lactating women have historically relied on reproductive toxicology studies in animals.^200 201^ Animal studies are inadequate predictors of teratogenicity and toxicity in humans.^7 76 80 120 135 174 178^ They are particularly poor in understanding long-term developmental consequences for the fetus,^178^ thus leading to problematic interpretation.^76127 200^,^202^ Animal studies can be time-intensive as they involve multiple sub-studies and require testing different dose levels per species.^202^ They are typically conducted late in the drug development process, with results often not finalised until after Phase 3 clinical trials in non-pregnant populations.^202 203^ The lengthy timeline and late initiation of such studies have contributed to exclusionary practices based on poor evidence for the safety and efficacy of the pharmaceutical products in pregnancy.^159 202^

### Designing studies for optimal safety and efficacy

Randomised controlled trials (RCTs) are the gold standard for evaluating efficacy and safety of pharmaceutical products,^82 179 204^ and optimal drug dosing in pregnancy.^130 140^ However, conducting RCTs with pregnant and lactating women is infeasible in the absence of preliminary safety data on fetal teratogenicity of investigational products.^70 82 85 131 137 160 190^ This data unavailability has led to differing recommendations regarding whether and at what stage to include pregnant and lactating women in clinical trials,^89 100 110 141 178^ and placebo use in this population.^70 82 85 137 190^ Selecting appropriate trial endpoints is crucial but challenging as they must capture outcomes related to drug safety and efficacy, and pregnancy-related physiological changes.^7 205^

### The challenges of detecting adverse events

Detecting adverse events in clinical trials is essential for determining whether pharmaceutical products are safe or not. To do this, researchers must define what constitutes an adverse event^128 150 189 200^ and compare those observed events against background rates.^94113 206^,^208^ This process poses several challenges. First, there is no standardised set of biomarkers for adverse events, and validating any such biomarkers would require exceptionally large studies.^128 200^ In low- and middle-income countries (LMICs), this is compounded by unavailability of standardised case definitions that, for example, distinguish stillbirths from miscarriages, or fetal growth restriction from preterm birth.^150 189^ Diagnostic limitations make it particularly challenging to identify adverse events in the fetus,^150 178 189 200 209^ and these challenges are exacerbated in LMICs due to inequalities in access to diagnostics.^209^

Having reliable data on specific diseases or events is crucial to determining adverse events. Both high-income countries (HICs) and LMICs experience difficulties from lack or sporadic availability or unreliability of these data leading to an inability to determine adverse events.^94 113 150 189 206 207^ In LMICs, poor data availability may also result from challenges experienced by local researchers in publishing their work due to methodological issues.^77^ Improving research infrastructure, including human resources, in LMICs is required not only to boost data availability,^122 176^ but also because data from HICs are less likely to be generalised to LMICs due to local or regional sociocultural determinants.^77 143^

### Recommendations to overcome exclusionary practices and promote safe inclusion

**Figure 2:**
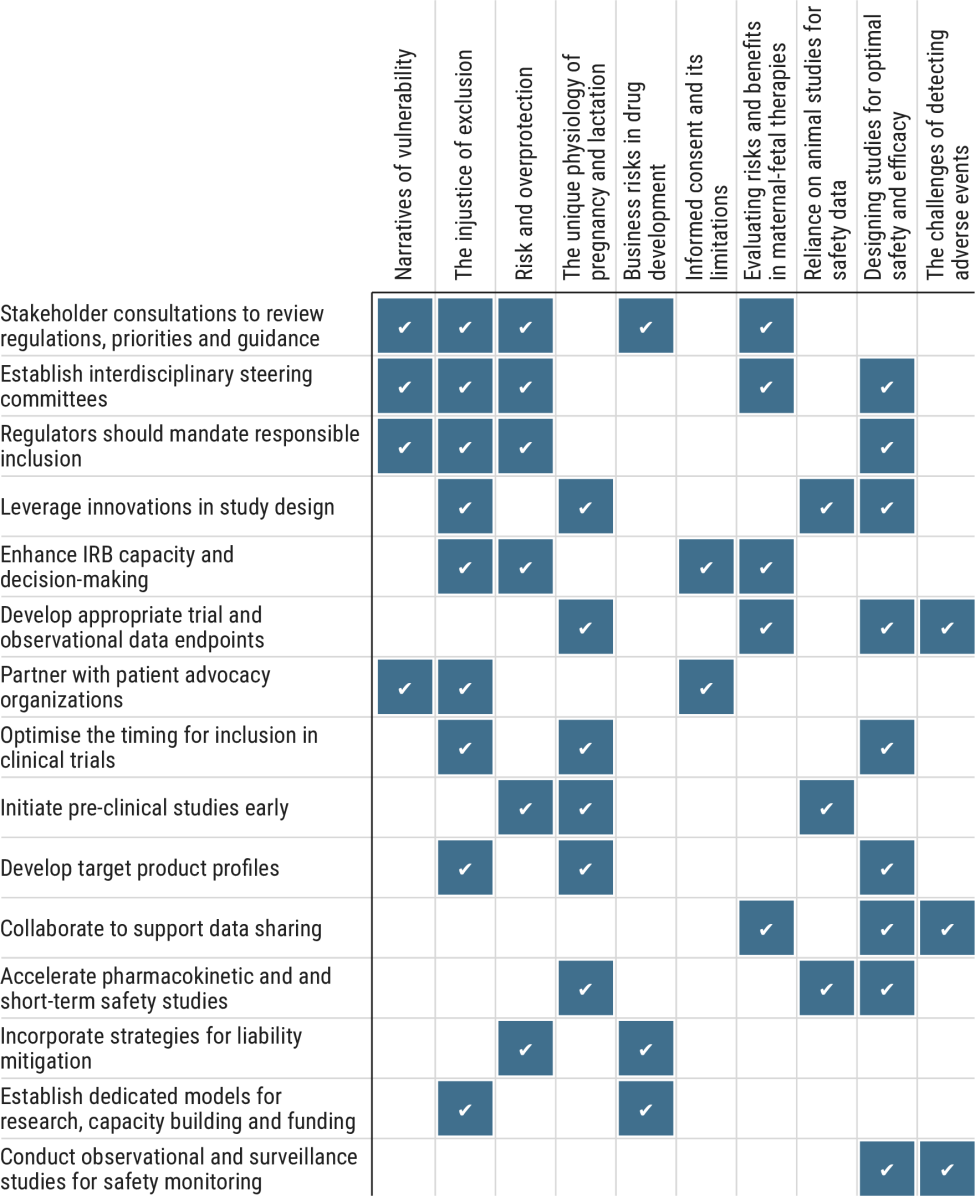
Alignment of recommendations with themes

Box 2 outlines recommendations from the literature for improving responsible inclusion in clinical trials, and figure 2 presents the alignment of these recommendations with review themes (for detailed recommendations, see online supplemental file 5: recommendations). Regulators must mandate scientific justification for exclusion. Early preclinical and developmental and reproductive toxicology studies are essential to provide data on placental drug transfer, fetal exposure and mechanism of drug action in the pregnant body. Vaccine and drug developers must ensure safety and efficacy for pregnancy and lactation in their target product profiles. Moving towards responsible inclusion requires developing standardised endpoints, operationalising ‘minimal risks’ across contexts and leveraging innovations (eg, adaptive trials) to optimise sample sizes and dosing. Increased funding is essential, including in support of research networks for sharing expertise, resources and data. Incentives for pharmaceutical companies to mitigate risk concerns may motivate their involvement.

Box 2 **Recommendations in brief**

1. Regulators should mandate inclusion of pregnant women in clinical trials, with scientific justification for any exclusions.^74 90 100 180^
2. Initiate preclinical studies, including Developmental and Reproductive Toxicology (DART) studies early in the clinical development of pharmaceutical products.^59 71 98 115 133 136 141 175 178 230^
3. Develop target product profiles with regulators during preclinical phases prioritising delivery systems and immune-boosting components that are safe and effective during pregnancy.^175^ ^228^
4. Optimise the timing for inclusion in clinical trials based on balance between the benefits and limitations of each option, tailoring trial design to the impact on pregnant and non-pregnant individuals, and, if necessary, employing separate or subgroup trials.^100 107 110 115 136 141 178^ ^228^
5. Accelerate pharmacokinetic and short-term safety studies by integrating innovative and complementary approaches early in drug development, ensuring data are available for licensure submissions, with comprehensive sampling across all pregnancy stages and distinct analysis for pregnant vs lactating women.^20 62 71 130 136 143 172 176 180 202 231 232^
6. Establish interdisciplinary steering committees to support responsible inclusion by guiding trial methodology and conduct for pregnant populations, with maternal-fetal medicine specialists providing direction and risk management during the trials.^102 180 205^
7. Leverage innovative study designs such as platform trials or adaptive design trials that allow for flexibility in adding or removing treatment arms or adjustments to sample size or intervention dosing.^62 98 130 133 204^
8. Develop appropriate trial and observational data endpoints by establishing and applying standardised case definitions for obstetric and neonatal outcomes, and training health workers to accurately identify and report congenital anomalies.^95 113 150 204 205 209^
9. Initiate stakeholder consultations to review regulations, set priorities and interpret guidance, including by achieving consensus on the definitions of ‘minimal risk’ and ‘direct benefit’ across different stages of pregnancy.^60 92 99 109^
10. Conduct observational and surveillance studies for safety monitoring by using opportunistic pharmacokinetic studies, cohort registries, case-control surveillance studies and population-pharmacokinetic studies to gather safety data.^26 60 62 92 113 115 118 127 128 134 173 204 207^ ^228^
11. Partner with patient advocacy organisations to optimise study design, enhance recruitment and retention, and improve data collection accuracy and completeness.^7 68 71^
12. Establish dedicated models for research, capacity strengthening and funding for drug and vaccine development targeting pregnant and lactating populations, ensuring streamlined regulatory pathways, incentives, expert workforce expansion, global and research networks, and robust training to enhance ethical, legal and infrastructural capacity.^29 60 62 89 92 98 99 107 128 134 136 204^
13. Incorporate strategies for liability mitigation to incentivise greater involvement of pharmaceutical companies in drug development for pregnant and lactating populations.^89 90 103^ ^228^
14. Enhance ethics committee capacity and decision-making by providing accessible indexed guidance, clear inclusion criteria, evidentiary standards and specialist consultation.^38 42 60 74 92 99 115 124 207 220^
15. Collaborate to support data sharing by strengthening advocacy networks, developing adaptable global guidance and prioritising harmonised data collection—such as Global Alignment of Immunisation and Antiviral Guidance recommendations—across all settings, including low- and middle-income countries.^60 150^

## Discussion

We synthesised and mapped arguments for the inclusion and exclusion of pregnant and lactating populations in clinical research, bridging gaps created by discipline-specific debates. Our synthesis yielded 10 themes and 15 recommendations to address challenges. Findings demonstrate multiple obstacles to responsible inclusion, some requiring structural reforms. Recommended changes include regulatory overhaul,^74 90 100 180^ reallocation of research funding,^29 60 62 89^ multi-site research networks^62 92 98 128 136 204^ and a deliberate shift to prioritising the unique therapeutic needs of pregnant and lactating people. Insights from the first WHO consultation on clinical trials for advancement of maternal and perinatal health support our findings and recommendations.^210^ Similar changes have improved evidence-based development of paediatric medicines in the USA and Europe.^62211^,^213^

The injustice of exclusion was the most common theme, highlighting how exclusionary practices deprive pregnant and lactating people of the benefits of biomedical research, shifting risks of medicine and vaccine use to clinical settings. These exclusionary practices stem from narratives of vulnerability that were historically embedded in regulations. Despite regulatory updates that no longer classify pregnant women as vulnerable, evaluations of US federal and industry-funded clinical trials suggest that progress towards responsible inclusion remains unchanged.^214 215^

In practice, researchers and ethics committees face challenges to apply regulatory guidance and bioethics frameworks to make nuanced decisions about risk-benefit trade-offs to the maternal-fetal dyad. The concept of fetal patienthood,^158^ which suggests a separate clinician-fetus relationship, complicates these decisions and can limit autonomy of the pregnant person. Blanket exclusions of pregnant individuals from clinical trials, often without clear scientific justification, undermine autonomy by preventing individuals from having the option and making an informed choice about enrolment. This approach fails to acknowledge the interconnected health interests of the maternal-fetal dyad and is misaligned with decision-making practices after birth, where parents are the primary decision-makers when balancing interests within a family.^216 217^

Limited human safety data in pregnant and lactating populations also impedes decision-making. This perpetuates a harmful cycle, both driving and being driven by a protectionist approach of exclusion. This cycle is reinforced by the lack of comprehensive regulatory guidance on inclusion in early phase trials, and the desire to safeguard against potential reputational damage and financial repercussions arising from the possibility of adverse consequences. Additionally, regulatory requirements for placebo controls can render trials infeasible and ethically problematic when they require some participants to receive care that is below the established standard.^218^ The International Council for Harmonisation of Technical Requirements for Pharmaceuticals for Human Use plans to introduce a ‘global harmonised guideline’ to standardise scientific and regulatory considerations for responsible inclusion of pregnant and lactating people in clinical research.^219^

Existing approaches overlook the harms of unmedicated or poorly medicated disease, that vary by levels of disease burden across geographies and health system capacities. Shifting to a ‘risk in context’^125^ evaluation demands abandoning blanket protectionism through collaboration among funders, regulators, researchers, ethics committees, community-based advocates, bioethicists, and maternal-fetal medicine and other specialists.^92 205^ Such collaboration is essential for interpreting guidance, contextualising key definitions and assessing the trustworthiness, relevance and sufficiency of evidence to make decisions about balancing potential benefits against the likelihood and magnitude of potential harms. Encouraging publications that describe culturally safe adaptations of trial procedures^187^ and risk-benefit assessments in clinical trial inclusion^220^ will offer practical guidance for peers in the field. Multidisciplinary collaboration is also crucial to tackle challenges in adverse event detection, including the lack of standardised approaches to case definitions and adverse event identification, large sample sizes to detect rare events, and in some settings, inadequate data on background rates of fetal and maternal conditions. These challenges speak to the need for complementary observational and surveillance data, including pharmacovigilance studies,^221^ to track maternal and fetal outcomes and medicine use during pregnancy.

Maternal-fetal therapies present unique ethical considerations where the pregnant person must undergo procedural risks alongside potential psychosocial benefits for anticipated health benefits to the fetus.^171^ Amid growing abortion restrictions in the USA,^222^ key considerations include the timing of offering the experimental intervention relative to pregnancy continuation decisions and ensuring accessibility of abortion care for those who undergo interventions but later opt not to or cannot continue the pregnancy.^222 223^

Limited investment in therapeutics for pregnant and lactating populations highlights shortcomings of a market-driven approach to pharmaceutical development. Substantial costs associated with novel drug development, including comprehensive liability insurance, are serious financial impediments. While LMICs bear a disproportionate burden of maternal morbidity and mortality, they are not financially attractive markets for the development of novel medications and may lack research and development infrastructure. High development costs, elevated risks, potential reputational damage and limited financial returns create an environment where market forces fail to incentivise innovation.^224^ Overcoming these challenges requires regulatory mandates for early initiation of developmental and reproductive toxicology studies and development of target product profiles that meet the needs of pregnant and lactating populations.^17 18^ Financial disincentives can be overcome through intergovernmental and philanthropic partnerships that establish dedicated funding models for multi-site preclinical, clinical and surveillance research and capacity-building^225^,^227^ with financial risk mitigation strategies, such as compensation plans for research-related injuries.^90 224 228 229^

Our review has limitations and strengths. We did not include grey literature, which may have affected the breadth and recency of data for some themes. We excluded book chapters to manage the volume of data, which may have led to the omission of relevant literature, especially in bioethics where this is a common publication format. However, many articles included in our review were classified under bioethics, suggesting that we have captured key ideas in this field. Our review focused on pregnant and lactating populations, as they are often grouped together in literature on this topic. However, in future, we recommend better differentiation between these groups as they present distinct physiological, ethical and practical considerations. This separation would facilitate more targeted strategies for addressing the unique challenges of including each group in research. A key strength is the review’s interdisciplinary approach that bridges disciplinary silos, providing a broad view of the issues involved and potential solutions. These insights may not be apparent when examining this topic through a single disciplinary lens.

## Conclusion

This scoping review reveals intersecting challenges that impede safe inclusion of pregnant and lactating populations in clinical research. Despite recent policy shifts, tangible improvements in research practices are limited. Our findings suggest that fragmented strategies are unlikely to be effective at overcoming exclusionary practices. We propose the need for structural changes across the drug development pipeline and research ecosystem. These changes would require coordinated multi-stakeholder efforts and dedicated long-term funding to prioritise scientific rigour and actively engage with ethical considerations and practical realities to reconstruct the research landscape to support responsible inclusion.

## Supplementary material

10.1136/bmjph-2025-003423 online supplemental file 1

## Data Availability

All data relevant to the study are included in the article or uploaded as supplementary information.
