# Supplementary material for: Bridging disciplinary siloes: a scoping review on the inclusion and exclusion of pregnant and lactating populations in clinical research
Source: BMJ Public Health. 2026 Jan 14;4(1):e003423. doi: 10.1136/bmjph-2025-003423 (PMC12815038; doi:10.1136/bmjph-2025-003423)
Supplement: online supplemental file 1 [file bmjph-4-1-s001.docx]

## **Supplemental File 1. PRISMA-ScR checklist**

| **SECTION** | **ITEM** | **PRISMA-ScR CHECKLIST ITEM** | **REPORTED ON PAGE #** |  |
| --- | --- | --- | --- | --- |
| **TITLE** | | | | |
| Title | 1 | Identify the report as a scoping review. | 1 |  |
| **ABSTRACT** | | | | |
| Structured summary | 2 | Provide a structured summary that includes (as applicable): background, objectives, eligibility criteria, sources of evidence, charting methods, results, and conclusions that relate to the review questions and objectives. | 2 |  |
| **INTRODUCTION** | | | | |
| Rationale | 3 | Describe the rationale for the review in the context of what is already known. Explain why the review questions/objectives lend themselves to a scoping review approach. | 4 |  |
| Objectives | 4 | Provide an explicit statement of the questions and objectives being addressed with reference to their key elements (e.g., population or participants, concepts, and context) or other relevant key elements used to conceptualize the review questions and/or objectives. | 4 |  |
| **METHODS** | | | | |
| Protocol and registration | 5 | Indicate whether a review protocol exists; state if and where it can be accessed (e.g., a Web address); and if available, provide registration information, including the registration number. | 5 |  |
| Eligibility criteria | 6 | Specify characteristics of the sources of evidence used as eligibility criteria (e.g., years considered, language, and publication status), and provide a rationale. | 5 |  |
| Information sources* | 7 | Describe all information sources in the search (e.g., databases with dates of coverage and contact with authors to identify additional sources), as well as the date the most recent search was executed. | 5-6, Supplemental file 2 |  |
| Search | 8 | Present the full electronic search strategy for at least 1 database, including any limits used, such that it could be repeated. | 5-6, Supplemental file 2 |  |
| Selection of sources of evidence† | 9 | State the process for selecting sources of evidence (i.e., screening and eligibility) included in the scoping review. | 6 |  |
| Data charting process‡ | 10 | Describe the methods of charting data from the included sources of evidence (e.g., calibrated forms or forms that have been tested by the team before their use, and whether data charting was done independently or in duplicate) and any processes for obtaining and confirming data from investigators. | 6 |  |
| Data items | 11 | List and define all variables for which data were sought and any assumptions and simplifications made. | 5-6 |  |
| Critical appraisal of individual sources of evidence§ | 12 | If done, provide a rationale for conducting a critical appraisal of included sources of evidence; describe the methods used and how this information was used in any data synthesis (if appropriate). | n/a |  |
| Synthesis of results | 13 | Describe the methods of handling and summarizing the data that were charted. | 6-14 |  |
| **RESULTS** | | | | |
| Selection of sources of evidence | 14 | Give numbers of sources of evidence screened, assessed for eligibility, and included in the review, with reasons for exclusions at each stage, ideally using a flow diagram. | 6, Figure 1 |  |
| Characteristics of sources of evidence | 15 | For each source of evidence, present characteristics for which data were charted and provide the citations. | 6, Supplemental file 3 |  |
| Critical appraisal within sources of evidence | 16 | If done, present data on critical appraisal of included sources of evidence (see item 12). | n/a |  |
| Results of individual sources of evidence | 17 | For each included source of evidence, present the relevant data that were charted that relate to the review questions and objectives. | 6, Supplemental file 3, Figure 2 |  |
| Synthesis of results | 18 | Summarize and/or present the charting results as they relate to the review questions and objectives. | 6-14, Figure 2 |  |
| **DISCUSSION** | | | | |
| Summary of evidence | 19 | Summarize the main results (including an overview of concepts, themes, and types of evidence available), link to the review questions and objectives, and consider the relevance to key groups. | 15-17 |  |
| Limitations | 20 | Discuss the limitations of the scoping review process. | 17 |  |
| Conclusions | 21 | Provide a general interpretation of the results with respect to the review questions and objectives, as well as potential implications and/or next steps. | 17 |  |
| **FUNDING** | | | | |
| Funding | 22 | Describe sources of funding for the included sources of evidence, as well as sources of funding for the scoping review. Describe the role of the funders of the scoping review. | 18 |  |

Supplemental File 2: Search Strategies

Medline

| # | Searches |
| --- | --- |
| 1 | (pregnant or pregnancy or lactating or lactation or breastfeed* or breast feed*).ti,kf. |
| 2 | pregnant women/ or pregnancy/ or breast feeding/ or lactation/ |
| 3 | 1 or 2 |
| 4 | ((inclu* or exclu* or participat* or recruit* or enrol* or involvement or involving or eligib* or decision or barrier* or facilitat* or ethic* or consent or acceptability or vulnerab* or engag* or regulat* or ethic*) adj6 (pregnan* or lactating or lactation or breastfeed* or breast feed*) adj6 (trials or research or clinical studies)).ab. |
| 5 | clinical studies as topic/ or exp clinical trials as topic/ or biomedical research/ or research subjects/ |
| 6 | ((drug or drugs or pharmaceutical* or vaccine* or medication or medications) adj2 (development or approval or regulation or evaluation)).ti,kf,hw. |
| 7 | ((inclu* or exclu* or participat* or recruit* or enrol* or involvement or involving or eligib* or decision or barrier* or facilitat* or ethic* or consent or acceptability or vulnerab* or engag* or regulat* or ethic*) and (pregnan* or lactating or lactation or breastfeed* or breast feed* or women or gender) and (trials or trial or research or clinical studies)).ti,kf. |
| 8 | 5 or 6 |
| 9 | health knowledge attitudes practice/ |
| 10 | motivation/ |
| 11 | patient participation/ |
| 12 | patient selection/ |
| 13 | decision making/ |
| 14 | eligibility determination/ |
| 15 | acceptability/ |
| 16 | informed consent/ |
| 17 | risk factors/ |
| 18 | attitude to health/ |
| 19 | stakeholder participation/ |
| 20 | risk assessment/ |
| 21 | trust/ |
| 22 | ethics.fs. |
| 23 | ethics committees research/ |
| 24 | research design/ |
| 25 | 9 or 10 or 11 or 12 or 13 or 14 or 15 or 16 or 17 or 18 or 19 or 20 or 21 or 22 or 23 or 24 |
| 26 | (regulat* or challenge* or eligib* or decision or barrier* or facilitat* or ethic* or moral* or consent or acceptability or vulnerab* or willing* or determinant* or benefit* or factor or factors or qualitative or altruis* or opportunit* or represent* or underrepresent* or risk or risks or unfair or fair or fairness or "scientifically complex" or stakeholder* or concerns or "focus group*" or interview* or recommend* or incentive*).mp. |
| 27 | (perception* or perspective* or opinion* or view or views or viewpoint* or reasons or reason or attitude* or experience*).mp. |
| 28 | (policy or policies or guideline* or model* or initiative* or implementation or "pharmaceutical compan*" or "pharmaceutical industr*" or "drug compan*" or "drug industr*" or "research agenda" or classif* or reclassif* or rule or rules or law or laws or legal or legislation or governance or guidance or safety or assessment or "research design" or "study design" or "research subjects").mp. |
| 29 | ("presumptive exclusion" or "unfair exclusion").mp. |
| 30 | ((participat* or inclu* or recruit* or enrol* or involv* or engag*) adj5 (pregnan* or lactating or lactation or breastfeed* or "breast feed*")).mp. |
| 31 | 25 or 26 or 27 or 28 or 29 or 30 |
| 32 | (3 and 8) or 4 or 7 |
| 33 | 32 and 31 |
| 34 | (clinical trial or randomized controlled trial or controlled clinical trial or clinical trial protocol).pt. |
| 35 | 33 not 34 |

EMBASE

| # | Searches |
| --- | --- |
| 1 | exp *named groups by pregnancy/ or exp *pregnancy/ or exp *breast feeding/ or *lactation/ or (pregnant or pregnancy or lactating or lactation or breastfeed* or breast feed*).ti,kf. |
| 2 | ((inclu* or exclu* or participat* or recruit* or enrol* or involvement or involving or eligib* or decision or barrier* or facilitat* or ethic* or consent or acceptability or vulnerab* or engag* or regulat* or ethic*) adj6 (pregnan* or lactating or lactation or breastfeed* or breast feed*) adj6 (trials or research or clinical studies)).ab. |
| 3 | exp "clinical trial (topic)"/ or medical research/ or clinical research/ or drug research/ or research subject/ |
| 4 | ((drug or drugs or pharmaceutical* or vaccine* or medication or medications) adj2 (development or approval or regulation or evaluation)).ti,kf,hw. |
| 5 | ((inclu* or exclu* or participat* or recruit* or enrol* or involvement or involving or eligib* or decision or barrier* or facilitat* or ethic* or consent or acceptability or vulnerab* or engag* or regulat* or ethic*) and (pregnan* or lactating or lactation or breastfeed* or breast feed* or women or gender) and (trials or trial or research or clinical studies)).ti,kf. |
| 6 | 3 or 4 |
| 7 | attitude to health/ or attitude to pregnancy/ or patient attitude/ |
| 8 | exp motivation/ |
| 9 | patient participation/ |
| 10 | patient selection/ |
| 11 | decision making/ or patient decision making/ or exp medical decision making/ |
| 12 | eligibility/ or eligibility criteria/ or legal aspect/ |
| 13 | program acceptability/ |
| 14 | informed consent/ |
| 15 | exp risk/ |
| 16 | stakeholder engagement/ |
| 17 | trust/ |
| 18 | exp ethics/ |
| 19 | professional standard/ |
| 20 | methodology/ or experimental design/ or study design/ |
| 21 | 7 or 8 or 9 or 10 or 11 or 12 or 13 or 14 or 15 or 16 or 17 or 18 or 19 or 20 |
| 22 | (regulat* or challenge* or eligib* or decision or barrier* or facilitat* or ethic* or moral* or consent or acceptability or vulnerab* or willing* or determinant* or benefit* or factor or factors or qualitative or altruis* or opportunit* or represent* or underrepresent* or risk or risks or unfair or fair or fairness or "scientifically complex" or stakeholder* or concerns or "focus group*" or interview* or recommend* or incentive*).mp. |
| 23 | (perception* or perspective* or opinion* or view or views or viewpoint* or reasons or reason or attitude* or experience*).mp. |
| 24 | (policy or policies or guideline* or model* or initiative* or implementation or "pharmaceutical compan*" or "pharmaceutical industr*" or "drug compan*" or "drug industr*" or "research agenda" or classif* or reclassif* or rule or rules or law or laws or legal or legislation or governance or guidance or safety or assessment or "research design" or "study design" or "research subjects").mp. |
| 25 | ("presumptive exclusion" or "unfair exclusion").mp. |
| 26 | ((participat* or inclu* or recruit* or enrol* or involv* or engag*) adj5 (pregnan* or lactating or lactation or breastfeed* or "breast feed*")).mp. |
| 27 | 21 or 22 or 23 or 24 or 25 or 26 |
| 28 | clinical trial/ or randomized controlled trial/ or controlled clinical trial/ or clinical trial protocol/ |
| 29 | systematic review/ |
| 30 | (1 or 2) and 6 |
| 31 | 30 or 5 |
| 32 | 31 and 27 |
| 33 | 32 not (28 or 29) |
| 34 | limit 33 to conference abstract status |
| 35 | 33 not 34 |

Global Health

| # | Searches |
| --- | --- |
| 1 | pregnant women/ or pregnancy/ or exp breast feeding/ or lactation/ or human lactation/ |
| 2 | (pregnant or pregnancy or lactating or lactation or breastfeed* or breast feed*).ti,id. |
| 3 | 1 or 2 |
| 4 | clinical trials/ or randomized controlled trials/ or biomedical research/ or research/ |
| 5 | ((drug or drugs or pharmaceutical* or vaccine* or medication or medications) adj2 (development or approval or regulation or evaluation)).ti,hw. |
| 6 | 4 or 5 |
| 7 | ((inclu* or exclu* or participat* or recruit* or enrol* or involvement or involving or eligib* or decision or barrier* or facilitat* or ethic* or consent or acceptability or vulnerab* or engag* or regulat* or ethic*) adj6 (pregnan* or lactating or lactation or breastfeed* or breast feed*) adj6 (trials or research or clinical studies)).ab. |
| 8 | ((inclu* or exclu* or participat* or recruit* or enrol* or involvement or involving or eligib* or decision or barrier* or facilitat* or ethic* or consent or acceptability or vulnerab* or engag* or regulat* or ethic*) and (pregnan* or lactating or lactation or breastfeed* or breast feed* or women or gender) and (trials or trial or research or clinical studies)).ti,id. |
| 9 | 3 and 6 |
| 10 | motivation/ or incentives/ |
| 11 | exp participation/ |
| 12 | decision making/ |
| 13 | eligibility/ |
| 14 | acceptability/ |
| 15 | consent/ |
| 16 | stakeholders/ |
| 17 | ethics/ or bioethics/ or medical ethics/ |
| 18 | committees/ or advisory committees/ |
| 19 | (regulatory or regulations or decision or barrier* or facilitat* or ethic* or moral* or acceptability or vulnerab* or willing* or qualitative or altruis* or opportunit* or underrepresent* or unfair or fair or fairness or "scientifically complex" or stakeholder* or concerns or "focus group*" or incentive*).mp. |
| 20 | (challenge* or eligib* or consent or recommend*).ti,id,hw. |
| 21 | (represent* adj6 (pregnan* or breastfeed* or breast feed* or lactating or lactation or women or minorit* or gender*)).mp. [mp=abstract, title, original title, heading words, cabicodes words] |
| 22 | (perception* or perspective* or opinion* or views or viewpoint* or reasons or reason or experience or experiences).mp. |
| 23 | attitude*.ti,id,hw. |
| 24 | (policy or policies or initiative* or "pharmaceutical compan*" or "pharmaceutical industr*" or "drug compan*" or "drug industr*" or "research agenda" or rule or rules or law or laws or legal or legislation or governance or guidance or "research design" or "study design" or "research subjects").mp. |
| 25 | (guideline* or implementation or safety).ti,id,hw. |
| 26 | ("presumptive exclusion " or "unfair exclusion").mp. |
| 27 | ((participat* or inclusion or recruit or recruitment or enrol or enrolment or enrollment or involvement or involving or engage or engagement) adj4 (pregnan* or lactating or lactation or breastfeed* or "breast feed*")).mp. |
| 28 | 10 or 11 or 12 or 13 or 14 or 15 or 16 or 17 or 18 or 19 or 20 or 21 or 22 or 23 or 24 or 25 or 26 or 27 |
| 29 | 9 and 28 |
| 30 | 7 or 8 or 29 |

CINAHL

| # | Searches |
| --- | --- |
| 1 | ( (MH "Expectant Mothers") OR (MH pregnancy) OR (MH "pregnancy, high risk") OR (MH "breast feeding") OR (MH lactation) ) OR TI ( pregnant OR pregnancy OR lactating OR lactation OR breastfeed* OR "breast feed*" ) |
| 2 | AB ((inclu* or exclu* or participat* or recruit* or enrol* or involvement or involving or eligib* or decision or barrier* or facilitat* or ethic* or consent or acceptability or vulnerab* or engag* or regulat* or ethic*) N5 (pregnan* or lactating or lactation or breastfeed* or "breast feed*") N5 (trials or research or "clinical studies")) |
| 3 | (MH "clinical trials") OR (MH "randomized controlled trials") OR (MH "research, medical") OR (MH +"research subjects") OR (MH "clinical research") |
| 4 | TI ( (drug or drugs or pharmaceutical* or vaccine* or medication or medications) N1 (development or approval or regulation or evaluation) ) OR SU ( (drug or drugs or pharmaceutical* or vaccine* or medication or medications) N1 (development or approval or regulation or evaluation) ) |
| 5 | TI ( (inclu* or exclu* or participat* or recruit* or enrol* or involvement or involving or eligib* or decision or barrier* or facilitat* or ethic* or consent or acceptability or vulnerab* or engag* or regulat* or ethic*) and (pregnan* or lactating or lactation or breastfeed* or "breast feed*" or women or gender) and (trials or trial or research or "clinical studies") ) |
| 6 | S3 OR S4 |
| 7 | (S1 AND S6) OR S2 OR S5 |
| 8 | (MH "Attitude to Health") OR (MH "Health Knowledge") |
| 9 | (MH "Motivation") |
| 10 | (MH "Consumer Participation") |
| 11 | (MH "Patient Selection") OR (MH "Research subject recruitment") OR (MH "Research subject retention") |
| 12 | (MH "Decision Making") OR (MH "Decision Making, Patient") OR (MH "Decision Making, Clinical") OR (MH "Decision Making, Ethical") |
| 13 | (MH "Eligibility Determination") |
| 14 | (MH "Consent (Research)") OR (MH Consent) OR (MH "Patient Autonomy") |
| 15 | (MH "Risk Factors") |
| 16 | (MH "Attitude to Health") OR (MH "Attitude to Pregnancy") OR (MH "Attitude to Risk") OR (MH "Patient Attitudes") |
| 17 | (MH "Stakeholder Participation") |
| 18 | (MH "Risk Assessment") |
| 19 | (MH "Trust") |
| 20 | (MH "Ethics Committees") OR (MH "Institutional Review") |
| 21 | (MH "Study Design") OR (MH "Clinical Trials/MT/CL/ST/LJ") OR (MH "Randomized Controlled Trials/MT/LJ/ST/OG") |
| 22 | S8 OR S9 OR S10 OR S11 OR S12 OR S13 OR S14 OR S15 OR S16 OR S17 OR S18 OR S19 OR S20 OR S21 |
| 23 | regulat* or challenge* or eligib* or decision or barrier* or facilitat* or ethic* or moral* or consent or acceptability or vulnerab* or willing* or determinant* or benefit* or factor or factors or qualitative or altruis* or opportunit* or represent* or underrepresent* or risk or risks or unfair or fair or fairness or "scientifically complex" or stakeholder* or concerns or "focus group*" or interview* or recommend* or incentive* |
| 24 | perception* or perspective* or opinion* or view or views or viewpoint* or reasons or reason or attitude* or experience* |
| 25 | policy or policies or guideline* or model* or initiative* or implementation or "pharmaceutical compan*" or "pharmaceutical industr*" or "drug compan*" or "drug industr*" or "research agenda" or classif* or reclassif* or rule or rules or law or laws or legal or legislation or governance or guidance or safety or assessment or "research design" or "study design" or "research subjects" |
| 26 | "presumptive exclusion" or "unfair exclusion" |
| 27 | (participat* or inclu* or recruit* or enrol* or involv* or engag*) N4 (pregnan* or lactating or lactation or breastfeed* or "breast feed*") |
| 28 | S22 OR S23 OR S24 OR S25 OR S26 OR S27 |
| 29 | S7 AND S28 |
| 30 | PT ("clinical trial" OR "randomized controlled trial" OR "protocol") |
| 31 | PT "systematic review" |
| 32 | S29 NOT S30 |
| 33 | S32 NOT S31 |

SocIndex, Family & Society Studies Worldwide

| # | Searches |
| --- | --- |
| 1 | (inclu* or exclu* or participat* or recruit* or enrol* or involvement or involving or eligib* or decision or barrier* or facilitat* or ethic* or consent or acceptability or vulnerab* or engag* or regulat* or ethic*) N5 (pregnan* OR lactat* OR "breast feed*" OR breastfeed*) N5 (trials OR research) |
| 2 | ( pregnan* OR lactat* OR "breast feed*" OR breastfeed* ) AND ( "human research subjects" OR "patient selection" OR "patient participation" OR "medical experimentation on humans") |
| 3 | ( pregnan* OR lactat* OR "breast feed*" OR breastfeed* ) AND SU ( "clinical trials" OR "randomized controlled trials" OR "medical research") |
| 4 | (regulatory or regulations or decision or barrier* or facilitat* or ethic* or moral* or acceptability or vulnerab* or willing* or qualitative or altruis* or opportunit* or underrepresent* or unfair or fair or fairness or "scientifically complex" or stakeholder* or concerns or "focus group*" or incentive*) |
| 5 | TI ( challenge* or eligib* or consent or recommend* ) OR SU ( challenge* or eligib* or consent or recommend* ) OR KW ( challenge* or eligib* or consent or recommend* ) |
| 6 | represent* N5 (pregnan* or breastfeed* or breast feed* or lactating or lactation or women or minorit* or gender*) |
| 7 | perception* or perspective* or opinion* or views or viewpoint* or reasons or reason or experience or experiences |
| 8 | attitude* |
| 9 | policy or policies or initiative* or "pharmaceutical compan*" or "pharmaceutical industr*" or "drug compan*" or "drug industr*" or "research agenda" or rule or rules or law or laws or legal or legislation or governance or guidance or "research design" or "study design" or "research subjects" |
| 10 | TI ( guideline* or implementation or safety ) OR SU ( guideline* or implementation or safety ) OR KW ( guideline* or implementation or safety ) |
| 11 | "presumptive exclusion" or "unfair exclusion" |
| 12 | (participat* or inclusion or recruit or recruitment or enrol or enrolment or enrollment or involvement or involving or engage or engagement) N3 (pregnan* or lactating or lactation or breastfeed* or "breast feed*") |
| 13 | motivation or incentive* or participation or "decision making" or eligibility or acceptability or stakeholder* or ethic* or bioethic* |
| 14 | S4 OR S5 OR S6 OR S7 OR S8 OR S9 OR S10 OR S11 OR S12 OR S13 |
| 15 | S3 AND S14 |
| 16 | S1 OR S2 OR S15 |

Web of Science

| # | Searches |
| --- | --- |
| 1 | TI=(inclu* or exclu* or participat* or recruit* or enrol* or "involvement" or "involving" or eligib* or "decision" or barrier* or facilitat* or ethic* or "consent" or "acceptability" or vulnerab* or engag* or regulat* or ethic*) OR AK=(inclu* or exclu* or participat* or recruit* or enrol* or "involvement" or "involving" or eligib* or "decision" or barrier* or facilitat* or ethic* or "consent" or "acceptability" or vulnerab* or engag* or regulat* or ethic*) |
| 2 | TI=(pregnan* or "lactating" or "lactation" or breastfeed* or "breast feed*" or "women" or "gender") OR AK=(pregnan* or "lactating" or "lactation" or breastfeed* or "breast feed*" or "women" or "gender") |
| 3 | TI=("trials" or "trial" or "research" or "clinical studies") OR AK=("trials" or "trial" or "research" or "clinical studies") |
| 4 | #1 AND #2 AND #3 |
| 5 | TI=("pregnant" or "pregnancy" or "lactating" or "lactation" or breastfeed* or "breast feed*") OR AK=("pregnant" or "pregnancy" or "lactating" or "lactation" or breastfeed* or "breast feed*") |
| 6 | TI=(("drug" or "drugs" or pharmaceutical* or vaccine* or "medication" or "medications") NEAR/1 ("development" or "approval" or "regulation" or "evaluation")) OR AK=(("drug" or "drugs" or pharmaceutical* or vaccine* or "medication" or "medications") NEAR/1 ("development" or "approval" or "regulation" or "evaluation")) |
| 7 | TI=("trials" OR "biomedical research" OR "drug research") OR AK=("trials" OR "biomedical research" OR "drug research") |
| 8 | #5 AND (#6 or #7) |
| 9 | AB=((inclu* or exclu* or participat* or recruit* or enrol* or "involvement" or "involving" or eligib* or "decision" or barrier* or facilitat* or ethic* or "consent" or "acceptability" or vulnerab* or engag* or regulat* or ethic*) NEAR/5 (pregnan* or "lactating" or "lactation" or breastfeed* or "breast feed*") NEAR/5 ("trials" or "research" or "clinical studies")) |
| 10 | #4 OR #8 OR #9 |
| 11 | TS=(regulat* or challenge* or eligib* or "decision" or barrier* or facilitat* or ethic* or moral* or "consent" or "acceptability" or vulnerab* or willing* or determinant* or benefit* or "factor" or "factors" or "qualitative" or altruis* or opportunit* or represent* or underrepresent* or "risk" or "risks" or "unfair" or "fair" or "fairness" or "scientifically complex" or stakeholder* or "concerns" or "focus group*" or interview* or recommend* or incentive*) |
| 12 | TS=(perception* or perspective* or opinion* or "view" or" views" or viewpoint* or "reasons" or "reason" or attitude* or experience*) |
| 13 | TS=("policy" or "policies" or guideline* or model* or initiative* or "implementation" or "pharmaceutical compan*" or "pharmaceutical industr*" or "drug compan*" or "drug industr*" or "research agenda" or classif* or reclassif* or "rule" or "rules" or "law" or "laws" or "legal" or "governance" or "guidance" or "safety" or "assessment" or "research design" or "study design" or "research subjects") |
| 14 | TS=("presumptive exclusion" or "unfair exclusion") |
| 15 | TS=((participat* or inclu* or recruit* or enrol* or involv* or engag*) NEAR/4 (pregnan* or "lactating" or "lactation" or breastfeed* or "breast feed*")) |
| 16 | TS=("knowledge" or "attitude*" OR "motivation" OR "selection" OR "trust" or committee*) |
| 17 | #11 OR #12 OR #13 OR #14 OR #15 OR #16 |
| 18 | #10 AND #17(Exclude – Document Types: Meeting Abstract) |

Scopus

Scopus search strategy was based on the Web of Science search

Supplemental File 3: PRISMA flowchart depicting search and selection process

**Identification**

**Screening**

**40,153** records identified from database searching

**13,869** duplicates removed

**26,284** titles and abstracts screened

**25,689 records** irrelevant

**595** full-texts assessed for eligibility

**407** full-texts excluded

**176 topic is not relevant to scoping review aim**

**94** not primary research

**83** not clinical or therapeutic interventional research

**53 population addressed is not pregnant or lactating women**

**1** no full-text

**188 papers**

**Included**

Supplemental File 4: Characteristics of individual papers

| **No** | **Author (Year of Publication)** | **Publication language** | **Disciplines** | **WHO region (countries)** | **Record type** | **Area of research** | **Focal population** |
| --- | --- | --- | --- | --- | --- | --- | --- |
| 1 | Abbas-Hanif (2021) | English | Pharmacology | The Americas (United States) | Review (Not specified) | Infectious diseases (COVID-19) | Pregnant women and/or the fetus |
| 2 | Abrams (2022) | English | Pharmacology; Epidemiology | Not specified/Not applicable | Commentary | Infectious diseases (Antiretrovirals) | Pregnant women and/or the fetus |
| 3 | Abtroun (2019) | French | Bioethics | Not specified/Not applicable | Commentary | Not specified | Pregnant women and/or the fetus |
| 4 | Adams (2007) | English | Bioethics | Western Pacific (China) | Other (lessons learned) | Complications of pregnancy and childbirth (Postpartum hemorrhage) | Pregnant women and/or the fetus |
| 5 | Adu-Bonsaffoh (2020) | English | Epidemiology; Bioethics; R&D | Africa (Ghana) | Review (Not specified) | Complications of pregnancy and childbirth (Preterm birth) | Pregnant women and/or the fetus |
| 6 | Alirol (2017) | English | Bioethics; Epidemiology | Europe (Switzerland); Africa (Kenya, Gabon) | Other (debate) | Infectious diseases (Ebola) | Pregnant women and/or the fetus |
| 7 | Allesee (2011) | English | Bioethics; Law and regulation | The Americas (United States) | Conceptual article | Not specified | Pregnant women and/or the fetus |
| 8 | Anderson (2012) | English | Bioethics | The Americas (United States) | Conceptual article | Complications of pregnancy and childbirth (Pregnant women with a history of venous thromboembolism and/or thrombophilia) | Pregnant women and/or the fetus |
| 9 | Anger (2008) | English | Pharmacology | Not specified/Not applicable | Other (report) | Not specified | Pregnant women and/or the fetus |
| 10 | Ballantyne (2016) | English | Bioethics; Law and regulation | Europe (New Zealand) | Opinion/Viewpoint | Not specified | Pregnant women and/or the fetus |
| 11 | Basaia (2014) | French | Bioethics; Law and regulation | Europe (France) | Review (Not specified) | Not specified | Pregnant women and/or the fetus |
| 12 | Baylis (2010) | English | Bioethics; Epidemiology | Not specified/Not applicable | Opinion/Viewpoint | Not specified | Pregnant women and/or the fetus |
| 13 | Baylis (2012) | English | Bioethics; Pharmacology; Epidemiology | Not specified/Not applicable | Review (Not specified) | Not specified | Pregnant women and/or the fetus |
| 14 | Baylis (2010) | English | Bioethics | The Americas (Canada) | Commentary | Not specified | Pregnant women and/or the fetus |
| 15 | Beigi (2021) | English | Bioethics; Pharmacology; Epidemiology | Not specified/Not applicable | Commentary | Infectious diseases (COVID-19) | Pregnant women and/or the fetus |
| 16 | Beigi (2013) | English | Law and regulation; Pharmacology; Epidemiology | Not specified/Not applicable | Editorial | Not specified | Pregnant women and/or the fetus |
| 17 | Bianchi (2021) | English | Bioethics; Pharmacology | The Americas (United States) | Opinion/Viewpoint | Infectious diseases (COVID-19) | Pregnant and lactating women |
| 18 | Biggio (2020) | English | Bioethics; Law and regulation | The Americas (United States) | Review (Not specified) | Not specified | Pregnant women and/or the fetus |
| 19 | Brandon (2009) | English | Bioethics; Law and regulation; Epidemiology; R&D | The Americas (United States) | Review (Not specified) | Mental health (Inclusion of pregnant women in psychiatric drug studies) | Pregnant and lactating women |
| 20 | Briggs (2015) | English | Pharmacology | Not specified/Not applicable | Opinion/Viewpoint | Not specified | Pregnant women and/or the fetus |
| 21 | Brummel (2022) | English | Pharmacology; Epidemiology | Not specified/Not applicable | Commentary | Infectious diseases (Antiretrovirals) | Pregnant women and/or the fetus |
| 22 | Burnham (2022) | English | Law and regulation; Pharmacology; Epidemiology; R&D | The Americas (United States) | Review (Not specified) | Not specified | Pregnant and lactating women |
| 23 | Caritis (2021) | English | Pharmacology | Not specified/Not applicable | Review (Not specified) | Not specified | Pregnant and lactating women |
| 24 | Carnovale (2021) | English | Pharmacology | Not specified/Not applicable | Review (Not specified) | Not specified | Pregnant and lactating women |
| 25 | Chappell (2021) | English | Pharmacology | Not specified/Not applicable | Commentary | Not specified | Pregnant and lactating women |
| 26 | Charo (1993) | English | Law and regulation | The Americas (United States) | Conceptual article | Not specified | Pregnant women and/or the fetus |
| 27 | Chervenak (1999) | English | Bioethics | Not specified/Not applicable | Opinion/Viewpoint | Fetal/newborn research (Not specified) | Pregnant women and/or the fetus |
| 28 | Chervenak (2003) | English | Bioethics | Not specified/Not applicable | Opinion/Viewpoint | Fetal/newborn research (Not specified) | Pregnant women and/or the fetus |
| 29 | Chervenak (2004) | English | Bioethics | Not specified/Not applicable | Conceptual article | Fetal/newborn research (Not specified) | Pregnant women and/or the fetus |
| 30 | Chervenak (2011) | English | Bioethics; Law and regulation | Not specified/Not applicable | Conceptual article | Fetal/newborn research (Not specified) | Pregnant women and/or the fetus |
| 31 | Chwang (2014) | English | Bioethics; Law and regulation | Not specified/Not applicable | Conceptual article | Not specified | Pregnant women and/or the fetus |
| 32 | Clayden (2022) | English | Bioethics; Epidemiology | Not specified/Not applicable | Opinion/Viewpoint | Infectious diseases (HIV) | Pregnant and lactating women |
| 33 | Colbers (2019) | English | Pharmacology | Not specified/Not applicable | Opinion/Viewpoint | Infectious diseases (HIV) | Pregnant and lactating women |
| 34 | Costantine (2020) | English | Bioethics | Not specified/Not applicable | Commentary | Infectious diseases (COVID-19) | Pregnant women and/or the fetus |
| 35 | Cottrell (2015) | English | Pharmacology | Not specified/Not applicable | Review (Not specified) | Complications of pregnancy and childbirth (Preeclampsia); Fetal/newborn research (Fetal growth) | Pregnant women and/or the fetus |
| 36 | Coverdale (2008) | English | Bioethics; Epidemiology | Not specified/Not applicable | Review (systematic review) | Mental health (Anetanal depression) | Pregnant women and/or the fetus |
| 37 | D'Angio (2023) | English | Bioethics | Not specified/Not applicable | Commentary | Not specified | Pregnant women and/or the fetus |
| 38 | David (2021) | English | Bioethics; Pharmacology; Epidemiology; R&D | Not specified/Not applicable | Review (Not specified) | Complications of pregnancy and childbirth (Not specified) | Pregnant women and/or the fetus |
| 39 | David (2022) | English | Law and regulation; Pharmacology; Epidemiology | Not specified/Not applicable | Review (Not specified) | Complications of pregnancy and childbirth (Not specified) | Pregnant women and/or the fetus |
| 40 | Denne (2019) | English | Pharmacology | Not specified/Not applicable | Editorial | Not specified | Pregnant women and/or the fetus |
| 41 | deOliveira (2023) | Spanish | Bioethics; Epidemiology | Not specified/Not applicable | Review (Not specified) | Infectious diseases (Not specified) | Pregnant women and/or the fetus |
| 42 | Deprest (2011) | English | Bioethics | Not specified/Not applicable | Opinion/Viewpoint | Fetal/newborn research (Not specified) | Pregnant women and/or the fetus |
| 43 | deWert (2017) | English | Bioethics | Not specified/Not applicable | Conceptual article | Fetal/newborn research (Fetal therapy for down syndrome) | Pregnant women and/or the fetus |
| 44 | Dhumale (2017) | English | Bioethics | India (South-East Asia) | Commentary | Complications of pregnancy and childbirth (Not specified) | Pregnant women and/or the fetus |
| 45 | Divala (2015) | English | Epidemiology; Bioethics | Africa (Malawi) | Other (lessons learned) | Infectious diseases (Not specified) | Pregnant women and/or the fetus |
| 46 | Donley (2015) | English | Bioethics; Law and regulation | The Americas (United States) | Conceptual article | Not specified | Pregnant women and/or the fetus |
| 47 | Eckenwiler (1999) | English | Bioethics; Law and regulation | Not specified/Not applicable | Conceptual article | Not specified | Pregnant women and/or the fetus |
| 48 | Eckenwiler (2001) | English | Bioethics | Not specified/Not applicable | Conceptual article | Not specified | Pregnant women and/or the fetus |
| 49 | Eckert (2020) | English | Epidemiology | Not specified/Not applicable | Review (Not specified) | Infectious diseases (Not specified) | Pregnant women and/or the fetus |
| 50 | Edwards (2020) | English | Bioethics; Epidemiology | Not specified/Not applicable | Other (report) | Infectious diseases (Ebola) | Pregnant women and/or the fetus |
| 51 | Eke (2020) | English | Pharmacology | Not specified/Not applicable | Opinion/Viewpoint | Infectious diseases (HIV) | Pregnant and lactating women |
| 52 | ElGaaloul (2022) | English | Pharmacology; Epidemiology | Not specified/Not applicable | Commentary | Infectious diseases (Malaria) | Pregnant women and/or the fetus |
| 53 | Elias (1983) | English | Bioethics; Law and regulation | The Americas (United States) | Review (Not specified) | Fetal/newborn research (Fetal surgery) | Pregnant women and/or the fetus |
| 54 | Faden (2017) | English | Bioethics; Epidemiology | Not specified/Not applicable | Commentary | Infectious diseases (Zika virus) | Pregnant women and/or the fetus |
| 55 | Fairlie (2019) | English | Pharmacology | Not specified/Not applicable | Commentary | Infectious diseases (HIV) | Pregnant and lactating women |
| 56 | Farrell (2020) | English | Bioethics; Epidemiology | Not specified/Not applicable | Conceptual article | Infectious diseases (COVID-19) | Pregnant women and/or the fetus |
| 57 | Farrell (2022) | English | Bioethics | The Americas (United States) | Commentary | Fetal/newborn research (Not specified) | Pregnant women and/or the fetus |
| 58 | Filbey (2022) | English | Bioethics; Pharmacology; Epidemiology | Not specified/Not applicable | Review (Not specified) | Non-communicable disease (Cardiovascular disease) | Pregnant and lactating women |
| 59 | Fisk (2008) | English | R&D | Not specified/Not applicable | Review (Not specified) | Not specified | Pregnant women and/or the fetus |
| 60 | Fletcher (1985) | English | Bioethics; Law and legislation | The Americas (United States) | Conceptual article | Fetal/newborn research (Not specified) | Pregnant women and/or the fetus |
| 61 | Fletcher (1992) | English | Bioethics; Law and legislation | The Americas (United States) | Review (Not specified) | Fetal/newborn research (Not specified) | Pregnant women and/or the fetus |
| 62 | Fletcher (1996) | English | Bioethics | Not specified/Not applicable | Conceptual article | Fetal/newborn research (Not specified) | Pregnant women and/or the fetus |
| 63 | Fletcher (1996) | English | Bioethics | Not specified/Not applicable | Conceptual article | Fetal/newborn research (Not specified) | Pregnant women and/or the fetus |
| 64 | Fry (2022) | English | Bioethics | Not specified/Not applicable | Commentary | Fetal/newborn research (Not specified) | Pregnant women and/or the fetus |
| 65 | Gates (1999) | English | Bioethics | Not specified/Not applicable | Commentary | Fetal/newborn research (Not specified) | Pregnant women and/or the fetus |
| 66 | Geller (2004) | English | Bioethics; Epidemiology | India(South-East Asia) | Other (special communication) | Complications of pregnancy and childbirth (Not specified) | Pregnant women and/or the fetus |
| 67 | Glantz (2000) | English | Bioethics; Law and legislation | The Americas (United States | Conceptual article | Not specified | Pregnant women and/or the fetus |
| 68 | Goldkind (2010) | English | Bioethics; Epidemiology | Not specified/Not applicable | Opinion/Viewpoint | Infectious diseases (H1N1 Influenza) | Pregnant women and/or the fetus |
| 69 | Gomes (2017) | English | Bioethics; Epidemiology | Not specified/Not applicable | Review (Not specified) | Infectious diseases (Ebola) | Pregnant women and/or the fetus |
| 70 | Gonzalez (2015) | English | Pharmacology | Not specified/Not applicable | Other (lessons learned) | Not specified | Pregnant women and/or the fetus |
| 71 | Gonzalez-Duarte (2019) | English | Bioethics | Not specified/Not applicable | Conceptual article | Not specified | Pregnant women and/or the fetus |
| 72 | Gorenberg (1992) | English | Bioethics; Law and regulation | The Americas (United States) | Conceptual article | Not specified | Pregnant women and/or the fetus |
| 73 | Graham (2019) | English | Bioethics | Not specified/Not applicable | Review (Not specified) | Infectious diseases (Ebola) | Pregnant women and/or the fetus |
| 74 | Greupink (2022) | English | Pharmacology | Not specified/Not applicable | Commentary | Infectious diseases (HIV) | Pregnant and lactating women |
| 75 | Gupta (2019) | English | Epidemiology | Not specified/Not applicable | Review (Not specified) | Infectious diseases (Tuberculosis) | Pregnant and lactating women |
| 76 | Heath (2020) | English | Pharmacology; Epidemiology | Not specified/Not applicable | Commentary | Infectious diseases (COVID-19( | Pregnant and lactating women |
| 77 | Helmreich (2007) | English | Bioethics; Law and regulation | The Americas (United States, Canada) | Commentary | Not specified | Pregnant women and/or the fetus |
| 78 | Hendriks (2022) | English | Bioethics | The Americas (United States) | Conceptual article | Fetal/newborn research (Not specified) | Pregnant women and/or the fetus |
| 79 | Heyrana (2018) | English | Bioethics; Law and regulation | Not specified/Not applicable | Opinion/Viewpoint | Non-communicable disease (Zika virus); Infectious diseases (HIV) | Pregnant women and/or the fetus |
| 80 | Hoffman (2021) | English | Bioethics; Pharmacology; Epidemiology | Not specified/Not applicable | Opinion/Viewpoint | Infectious diseases (HIV) | Pregnant and lactating women |
| 81 | Ibuki (2022) | English | Bioethics | Not specified/Not applicable | Commentary | Fetal/newborn research (Not specified) | Pregnant women and/or the fetus |
| 82 | Illamola (2018) | English | Pharmacology; Epidemiology; Law and regulation | Not specified/Not applicable | Commentary | Not specified | Pregnant and lactating women |
| 83 | Iltis (2011) | English | Bioethics | Not specified/Not applicable | Commentary | Fetal/newborn research (Not specified) | Pregnant women and/or the fetus |
| 84 | Jaffe (2021) | English | Bioethics; Law and regulation | The Americas (United States) | Commentary | Infectious diseases (COVID-19) | Pregnant women and/or the fetus |
| 85 | Jhaveri (2021) | English | Bioethics; Pharmacology; R&D | The Americas (United States) | Review (systematic review) | Infectious diseases (Hepatitis C) | Pregnant women and/or the fetus |
| 86 | Johansson (2020) | English | Bioethics | Not specified/Not applicable | Conceptual article | Not specified | Pregnant women and/or the fetus |
| 87 | Jorgensen (2022) | English | Bioethics; Epidemiology; R&D | Not specified/Not applicable | Review (systematic review) | Not specified | Pregnant and lactating women |
| 88 | Kaposy (2012) | English | Bioethics; R&D | Not specified/Not applicable | Conceptual article | Not specified | Pregnant women and/or the fetus |
| 89 | Kaposy (2011) | English | Bioethics; Law and regulation | The Americas (United States) | Conceptual article | Not specified | Pregnant women and/or the fetus |
| 90 | Kass (1996) | English | Bioethics; Law and regulation | Not specified/Not applicable | Conceptual article | Infectious diseases (HIV) | Pregnant women and/or the fetus |
| 91 | Kaye (2019) | English | Bioethics; Pharmacology; Law and regulation | Not specified/Not applicable | Review (Not specified) | Complications of pregnancy and childbirth (Not specified) | Pregnant and lactating women |
| 92 | Kazma (2023) | English | Bioethics; Pharmacology; Law and regulation | Not specified/Not applicable | Review (Not specified) | Not specified | Pregnant women and/or the fetus |
| 93 | Keitt (2003) | English | Bioethics; Law and regulation | The Americas (United States) | Conceptual article | Not specified | Pregnant women and/or the fetus |
| 94 | Knaapen (2020) | English | Law and regulation | Europe (Not specified) | Review (Not specified) | Not specified | Pregnant women and/or the fetus |
| 95 | Kochhar (2017) | English | Bioethics; Epidemiology | Not specified/Not applicable | Commentary | Not specified | Pregnant women and/or the fetus |
| 96 | Korth-Bradley (2016) | English | Pharmacology | Not specified/Not applicable | Commentary | Not specified | Pregnant and lactating women |
| 97 | Krubiner (2017) | English | Bioethics | Not specified/Not applicable | Commentary | Not specified | Pregnant women and/or the fetus |
| 98 | LaCourse (2020) | English | Law and regulation | The Americas (United States) | Opinion/Viewpoint | Infectious diseases (COVID-19) | Pregnant and lactating women |
| 99 | Ledward (2011) | English | Bioethics | Not specified/Not applicable | Conceptual article | Not specified | Pregnant women and/or the fetus |
| 100 | Levine (2004) | English | Bioethics | Not specified/Not applicable | Conceptual article | Not specified | Pregnant women and/or the fetus |
| 101 | Liaschenko (2011) | English | Bioethics | Not specified/Not applicable | Commentary | Not specified | Pregnant women and/or the fetus |
| 102 | Little (2017) | English | Bioethics | Not specified/Not applicable | Other (Conference Proceedings) | Not specified | Pregnant women and/or the fetus |
| 103 | Lucas (2021) | English | Bioethics | Not specified/Not applicable | Review (Not specified) | Infectious diseases (COVID-19) | Pregnant women and/or the fetus |
| 104 | Lupton (2004) | English | Bioethics | Not specified/Not applicable | Commentary | Not specified | Pregnant women and/or the fetus |
| 105 | Lyerly (2019) | English | Bioethics | Not specified/Not applicable | Commentary | Infectious diseases (HIV) | Pregnant women and/or the fetus |
| 106 | Lyerly (2001) | English | Bioethics | Not specified/Not applicable | Conceptual article | Fetal/newborn research (Not specified) | Pregnant women and/or the fetus |
| 107 | Lyerly (2008) | English | Bioethics | Not specified/Not applicable | Review (Not specified) | Not specified | Pregnant women and/or the fetus |
| 108 | Lyerly (2011) | English | Bioethics | Not specified/Not applicable | Commentary | Not specified | Pregnant women and/or the fetus |
| 109 | Lyerly (2013) | English | Bioethics; Pharmacology | Not specified/Not applicable | Commentary | Not specified | Pregnant women and/or the fetus |
| 110 | Lyerly (2021) | English | Bioethics | Not specified/Not applicable | Commentary | Infectious diseases (HIV) | Pregnant women and/or the fetus |
| 111 | MacKay (2020) | English | Bioethics | Not specified/Not applicable | Conceptual article | Not specified | Pregnant women and/or the fetus |
| 112 | Macklin (2010) | English | Law and regulation | The Americas (United States); Europe (Not specified) | Commentary | Not specified | Pregnant women and/or the fetus |
| 113 | Malhame (2023) | English | Epidemiology | Not specified/Not applicable | Editorial | Infectious diseases (Not specified) | Pregnant women and/or the fetus |
| 114 | Malhame (2020) | English | Epidemiology; Pharmacology | Not specified/Not applicable | Opinion/Viewpoint | Infectious diseases (COVID-19) | Pregnant and lactating women |
| 115 | Malhotra (2021) | English | Bioethics | Not specified/Not applicable | Commentary | Infectious diseases (COVID-19) | Pregnant women and/or the fetus |
| 116 | Manca (2022) | English | Bioethics; Law and regulation | Not specified/Not applicable | Commentary | Infectious diseases (COVID-19) | Pregnant and lactating women |
| 117 | Mastroianni (1998) | English | Law and regulation | The Americas (United States) | Conceptual article | Infectious diseases (HIV) | Pregnant women and/or the fetus |
| 118 | Mastroianni (2017) | English | Law and regulation; R&D | The Americas (United States) | Review (Not specified) | Infectious diseases (Not specified) | Pregnant women and/or the fetus |
| 119 | Matsui (2015) | English | Bioethics; Law and regulation | Not specified/Not applicable | Review (Not specified) | Not specified | Pregnant women and/or the fetus |
| 120 | McCullough (2005) | English | Bioethics; Epidemiology; Law and regulation | Not specified/Not applicable | Conceptual article | Not specified | Pregnant women and/or the fetus |
| 121 | McCullough (2015) | English | Bioethics; Epidemiology; Law and regulation | Not specified/Not applicable | Review (Not specified) | Mental health (Psychosis) | Pregnant women and/or the fetus |
| 122 | Merton (1993) | English | Bioethics; Law and regulation | Not specified/Not applicable | Conceptual article | Not specified | Pregnant women and/or the fetus |
| 123 | Minkoff (1992) | English | Bioethics | The Americas (United States) | Conceptual article | Not specified | Pregnant women and/or the fetus |
| 124 | Miracle (2010) | English | Bioethics | Not specified/Not applicable | Review (Not specified) | Not specified | Pregnant women and/or the fetus |
| 125 | Morrell (1997) | English | Bioethics; Pharmacology | Not specified/Not applicable | Review (Not specified) | Non-communicable disease (Epilepsy) | Pregnant women and/or the fetus |
| 126 | Munoz (2022) | English | Law and regulation; Pharmacology; Epidemiology | Not specified/Not applicable | Opinion/Viewpoint | Infectious diseases (Not specified) | Pregnant women and/or the fetus |
| 127 | Munoz (2014) | English | Law and regulation; Epidemiology | Not specified/Not applicable | Editorial | Infectious diseases (Not specified) | Pregnant women and/or the fetus |
| 128 | Mutanga (2022) | English | Law and regulation; Epidemiology | The Americas (United States) | Review (Not specified) | Infectious diseases (Not specified) | Pregnant women and/or the fetus |
| 129 | Mystakidou (2009) | English | Bioethics | Not specified/Not applicable | Review (Not specified) | Infectious diseases (HIV) | Pregnant women and/or the fetus |
| 130 | Ngure (2017) | English | Bioethics | Africa (Kenya) | Case report | Infectious diseases (HIV) | Pregnant women and/or the fetus |
| 131 | Paris (2001) | English | Bioethics | The Americas (United States) | Conceptual article | Fetal/newborn research (Not specified) | Pregnant women and/or the fetus |
| 132 | Parkinson (1997) | English | Pharmacology | Western Pacific (Japan); The Americas (United States); Europe (Not specified) | Primary research (cross sectional study) | Not specified | Pregnant women and/or the fetus |
| 133 | Payne (2019) | English | Bioethics | The Americas (United States) | Conceptual article | Not specified | Pregnant women and/or the fetus |
| 134 | Peppin (2003) | English | Law and regulation; R&D | The Americas (United States, Canada) | Conceptual article | Mental health (Not specified) | Pregnant women and/or the fetus |
| 135 | Perry (2011) | English | Bioethics | Not specified/Not applicable | Conceptual article | Fetal/newborn research (Not specified) | Pregnant women and/or the fetus |
| 136 | Persampieri (2019) | English | Bioethics; Law and regulation | Not specified/Not applicable | Review (Not specified) | Not specified | Pregnant women and/or the fetus |
| 137 | Rasmussen (2014) | English | Epidemiology | Not specified/Not applicable | Review (Not specified) | Infectious diseases (Not specified) | Pregnant women and/or the fetus |
| 138 | Reitsma (2003) | English | Bioethics; Law and regulation | Not specified/Not applicable | Conceptual article | Fetal/newborn research (Maternal-fetal surgery) | Pregnant women and/or the fetus |
| 139 | Ren (2021) | English | Pharmacology; Law and regulation | Not specified/Not applicable | Review (Not specified) | Not specified | Pregnant and lactating women |
| 140 | Roberts (2015) | English | Law and regulation; Epidemiology | The Americas (United States) | Review (Not specified) | Infectious diseases (Not specified) | Pregnant women and/or the fetus |
| 141 | Rodrigues (2014) | English | Bioethics | Not specified/Not applicable | Conceptual article | Fetal/newborn research (Maternal-fetal surgery) | Pregnant women and/or the fetus |
| 142 | Roes (2018) | English | Bioethics; Epidemiology | Not specified/Not applicable | Other (Methodology) | Not specified | Pregnant women and/or the fetus |
| 143 | RosetBahmanyar (2021) | English | Law and regulation; Epidemiology; Pharmacology; R&D | Not specified/Not applicable | Opinion/Viewpoint | Complications of pregnancy and childbirth (Not specified) | Pregnant women and/or the fetus |
| 144 | Rothenberg (1996) | English | Bioethics; Law and regulation | The Americas (United States) | Conceptual article | Not specified | Pregnant women and/or the fetus |
| 145 | Saenz (2017) | English | Law and regulation | The Americas (United States) | Review (Not specified) | Not specified | Pregnant women and/or the fetus |
| 146 | Salas (2017) | Spanish | Bioethics | The Americas (Chile) | Case report | Complications of pregnancy and childbirth (Preterm birth) | Pregnant women and/or the fetus |
| 147 | Sandomire (1993) | English | Bioethics; Law and regulation | The Americas (United States) | Conceptual article | Not specified | Pregnant women and/or the fetus |
| 148 | Schonfeld (2013) | English | Bioethics | Not specified/Not applicable | Conceptual article | Not specified | Pregnant women and/or the fetus |
| 149 | Schwartz (2019) | English | Bioethics; R&D | Not specified/Not applicable | Opinion/Viewpoint | Infectious diseases (Ebola) | Pregnant women and/or the fetus |
| 150 | Schwartz (2019) | English | Bioethics | Africa (Congo, Dem. Rep.) | Opinion/Viewpoint | Infectious diseases (Ebola) | Pregnant women and/or the fetus |
| 151 | Schwartz (2018) | English | Pharmacology | Africa (Congo, Dem. Rep., West Africa region) | Opinion/Viewpoint | Infectious diseases (Zika virus) | Pregnant and lactating women |
| 152 | Schwarz (2005) | German | Law and regulation | Europe (Germany) | Review (Not specified) | Not specified | Pregnant and lactating women |
| 153 | Schwenzer (2008) | English | Bioethics | The Americas (United States) | Review (Not specified) | Infectious diseases (Respiratory disease) | Pregnant women and/or the fetus |
| 154 | Sengupta (2010) | English | Bioethics; Law and regulation | The Americas (United States) | Primary research (Qualitative research) | Infectious diseases (HIV) | Pregnant women and/or the fetus |
| 155 | Sewell (2022) | English | Bioethics; Law and regulation; Epidemiology; Pharmacology | The Americas (United States) | Other (Report) | Not specified | Pregnant women and/or the fetus |
| 156 | Shah (2022) | English | Bioethics; Law and regulation | The Americas (United States) | Editorial | Fetal/newborn research (Not specified) | Pregnant women and/or the fetus |
| 157 | Shamshirsaz (2019) | English | Bioethics | The Americas (United States) | Commentary | Fetal/newborn research (Not specified) | Pregnant women and/or the fetus |
| 158 | Sheppard (2016) | English | Bioethics; Law and regulation | Europe (United Kingdom, Germany, Sweden, Spain) | Primary research (Qualitative research) | Fetal/newborn research (Not specified) | Pregnant women and/or the fetus |
| 159 | Sheppard (2016) | English | Bioethics | Europe (United Kingdom) | Conceptual article | Fetal/newborn research (Not specified) | Pregnant women and/or the fetus |
| 160 | Sheppard (2016) | English | Bioethics; Law and regulation | Europe (United Kingdom) | Conceptual article | Fetal/newborn research (Not specified) | Pregnant women and/or the fetus |
| 161 | Singh (2022) | English | Bioethics; Epidemiology; Pharmacology; Law and regulation | Not specified/Not applicable | Opinion/Viewpoint | Infectious diseases (HIV) | Pregnant and lactating women |
| 162 | Spong (2018) | English | Epidemiology; Pharmacology; R&D | The Americas (United States) | Opinion/Viewpoint | Not specified | Pregnant and lactating women |
| 163 | Sportiello (2023) | English | Bioethics; Pharmacology; Law and regulation | Europe (Not specified) | Opinion/Viewpoint | Not specified | Pregnant and lactating women |
| 164 | Stock (2019) | English | Pharmacology; Epidemiology | Not specified/Not applicable | Review (Not specified) | Not specified | Pregnant women and/or the fetus |
| 165 | Strong (2011) | English | Bioethics; Law and regulation | The Americas (United States) | Review (Not specified) | Fetal/newborn research (Not specified) | Pregnant women and/or the fetus |
| 166 | Strong (2012) | English | Bioethics; Law and regulation | The Americas (United States) | Conceptual article | Not specified | Pregnant women and/or the fetus |
| 167 | Strong (2011) | English | Bioethics; Law and regulation | The Americas (United States) | Conceptual article | Fetal/newborn research (Not specified) | Pregnant women and/or the fetus |
| 168 | Strong (2011) | English | Bioethics; Law and regulation | The Americas (United States) | Conceptual article | Not specified | Pregnant women and/or the fetus |
| 169 | Strong (2011) | English | Bioethics | Not specified/Not applicable | Commentary | Fetal/newborn research (Not specified) | Pregnant women and/or the fetus |
| 170 | Theiler (2009) | English | Pharmacology; Epidemiology; R&D | Not specified/Not applicable | Opinion/Viewpoint | Infectious diseases (Antimicrobial therapy) | Pregnant women and/or the fetus |
| 171 | Thiele (2023) | English | Law and regulation; Bioethics | The Americas (United States) | Review (Not specified) | Not specified | Pregnant and lactating women |
| 172 | Thornton (2009) | English | Pharmacology; R&D | Not specified/Not applicable | Opinion/Viewpoint | Complications of pregnancy and childbirth (Not specified) | Pregnant women and/or the fetus |
| 173 | Turner (2019) | English | Bioethics; Law and regulation; Pharmacology; Epidemiology; R&D | Not specified/Not applicable | Review (Not specified) | Not specified | Pregnant women and/or the fetus |
| 174 | vanderGraaf (2018) | English | Bioethics; Law and regulation; Epidemiology | Not specified/Not applicable | Conceptual article | Not specified | Pregnant women and/or the fetus |
| 175 | vanderZande (2017) | English | Bioethics; Pharmacology; Law and regulation | Not specified/Not applicable | Review (Not specified) | Not specified | Pregnant women and/or the fetus |
| 176 | vanderZande (2021) | English | Bioethics | Not specified/Not applicable | Conceptual article | Not specified | Pregnant women and/or the fetus |
| 177 | vanLier (1986) | English | Bioethics | The Americas (United States) | Opinion/Viewpoint | Complications of pregnancy and childbirth (Not specified) | Pregnant women and/or the fetus |
| 178 | VanSpall (2021) | English | Epidemiology; Pharmacology | Not specified/Not applicable | Opinion/Viewpoint | Infectious diseases (COVID-19) | Pregnant and lactating women |
| 179 | Vernon (2006) | English | Bioethics | Europe (United Kingdom) | Commentary | Complications of pregnancy and childbirth (Not specified) | Pregnant women and/or the fetus |
| 180 | Waggoner (2022) | English | Bioethics; Law and regulation | The Americas (United States) | Review (Not specified) | Not specified | Pregnant women and/or the fetus |
| 181 | Welch (2015) | English | Bioethics; Law and regulation | The Americas (United States) | Review (Not specified) | Not specified | Pregnant women and/or the fetus |
| 182 | Weld (2021) | English | Bioethics; Law and regulation; Pharmacology; Epidemiology | Not specified/Not applicable | Review (Not specified) | Not specified | Pregnant and lactating women |
| 183 | Wendler (2020) | English | Bioethics; Law and regulation | The Americas (United States) | Conceptual article | Not specified | Pregnant women and/or the fetus |
| 184 | Wendler (1998) | English | Bioethics; Law and regulation | Not specified/Not applicable | Conceptual article | Not specified | Pregnant women and/or the fetus |
| 185 | White (2015) | English | Bioethics; Law and regulation | Not specified/Not applicable | Conceptual article | Not specified | Pregnant women and/or the fetus |
| 186 | Wild (2007) | German | Bioethics; Law and regulation | Europe (Germany) | Review (Not specified) | Not specified | Pregnant women and/or the fetus |
| 187 | Wild (2012) | English | Bioethics; Law and regulation | Europe (Germany) | Conceptual article | Not specified | Pregnant women and/or the fetus |
| 188 | Zimmerman (2015) | English | Bioethics; Law and regulation | Not specified/Not applicable | Review (Not specified) | Not specified | Pregnant women and/or the fetus |

Supplemental File 5: Recommendations

| **No** | **Recommendation** | **Sources** |
| --- | --- | --- |
| 1 | **Regulators should mandate inclusion**  Regulators should mandate the inclusion of pregnant women in clinical trials at appropriate stages and require follow-up studies for pharmaceutical safety. They should demand sound scientific justification for exclusion based on biological data, observational findings, or non-human primate trials. During health emergencies, regulators should mandate a systematic approach to include pregnant and lactating populations in vaccine trials. | 74,90,100,180 |
| 2 | **Initiate pre-clinical studies early**  Pre-clinical studies, including Developmental and Reproductive Toxicology (DART) studies, should start early in the clinical development of pharmaceutical products. These studies should assess placental transfer of drugs, fetal exposure, and effectiveness of the drug's mechanism of action during pregnancy. Results should be published promptly to support decision-making on testing in pregnant women. These data will help assess risks, rule out teratogenic effects and facilitate inclusion in Phase I, II, or IIb/III trials. Regulations should require the pharmaceutical industry to initiate these studies at the outset of or before initiating Phase 1 trials. | 59,71,98,115,133,136,141,175,178,228 |
| 3 | **Develop target product profiles**  For drug and vaccine development, define the target product profile with regulators during preclinical phases. Prioritise drug and vaccine candidates that use delivery systems (platforms) and immune-boosting components (adjuvants) that are safe and effective during pregnancy. | 175,228 |
| 4 | **Optimise the timing for inclusion in clinical trials**  Clinical trial design, including at which stage to involve pregnant women, depends on a balance between the benefits and limitations of each option, and how the intervention affects pregnant and non-pregnant individuals. Significant differences may require separate trials for pregnant women or subgroup analyses within larger trials. If effects are likely similar, post-marketing studies may suffice. Consult funders, scientific advisors, and stakeholders to establish robust research infrastructure early | 100,107,110,115,136,141,178,228 |
| 5 | **Accelerate pharmacokinetic and short-term safety studies**  Expedite pharmacokinetic and short-term safety studies to ensure results are available by the completion of Phase 3 trials in the general population for inclusion in licensure submissions to regulatory bodies. To accelerate these studies, innovative approaches can be adopted. These include ex vivo human cotyledon perfusion, in silico models, physiology-based pharmacokinetic (PBPK) modelling and population pharmacokinetics (PPPK) models. Additionally, small pharmacokinetics (PK) studies can be conducted alongside Phase II or III trials in non-pregnant adults. Other methods involve short-course (targeted) PK studies to assess PK properties of a drug administered in a continuous slow-release fashion, and micro-dosing studies. For selection of vaccine candidates for pregnant populations in outbreak contexts, pharmacokinetic studies should be initiated before and in-between outbreaks. Pharmacokinetic and short-term safety studies should proactively incorporate sampling throughout all stages of pregnancy and postpartum periods. Differentiate between pregnant and lactating women in study designs rather than grouping them under a single inclusion/exclusion criterion. | 20,62,71,130,136,143,172,176,180,202,229,230 |
| 6 | **Establish interdisciplinary steering committees to support responsible inclusion**  Form interdisciplinary trial steering committees to guide trial methodology and conduct for pregnant populations. Include maternal-fetal medicine specialists on these committees and trial teams to provide direction and manage risks during the trials. | 102,180,205 |
| 7 | **Leverage innovative study designs**  Consider innovative study designs for the conduct of clinical trials with pregnant women:  1) Platform Trials enable multi-arm studies, allowing quicker transitions from pharmacokinetic (PK) studies to Phase 3 randomised controlled trials (RCTs), and offering flexibility to add or remove treatment arms based on emerging evidence.  2) Adaptive Design Trials allow adjustments to sample size and dosing with provisions for early stopping due to superiority or futility, response-adaptive allocation, and the integration of multiple trial phases.  3) Interim Analyses throughout Phase III can assess safety and efficacy, helping to determine whether an early stop is warranted.  4) Multi-Site Study Protocols with broad eligibility criteria evaluating multiple drugs enhance efficiency of data collection on medication use during pregnancy. | 62,98,130,133,204 |
| 8 | **Develop appropriate trial and observational data endpoints**  Establish and/or apply standardised case definitions for obstetric and neonatal outcomes, including adverse events, for real-time monitoring in trials and observational studies. Train health workers to accurately identify and report congenital anomalies. Analyse adverse events as secondary outcomes in trials, both as composite endpoints and individual indicators to assess associations with experimental vaccines and treatments. | 95,113,150,204,205,209 |
| 9 | **Initiate stakeholder consultations to review regulations, set priorities, and interpret guidance**  Conduct stakeholder consultations globally to review regulations, guidelines, funding and research implementation for responsible inclusion of pregnant women in clinical research. Set research priorities for equitable trial representation, with key stakeholders involved in drug development and research. Achieve consensus on the definitions of "minimal risk" and "direct benefit" across different stages of pregnancy. Professional bodies should establish clear guidelines on "minimal risk" to assist ethics committees. Review past approaches that have been successful in managing risks in clinical trials, analysing the factors contributing to success, and understanding the legal frameworks that supported these efforts to guide future initiatives. | 60,92,99,109 |
| 10 | **Conduct observational and surveillance studies for safety monitoring**  Initiate post-marketing surveillance studies once a drug or vaccine enters the market, particularly for those lacking pre-marketing data. Use observational databases to track medication use in pregnancy and collect data on fetal outcomes, including for rare conditions where randomised controlled trials are not feasible. Use opportunistic pharmacokinetic studies, cohort registries, case-control surveillance studies, and population-pharmacokinetic studies to gather safety data. Develop robust data collection systems in preparation for pandemics, leveraging existing obstetrics monitoring frameworks and establishing pregnancy registries for vaccine pharmacovigilance, particularly in low- and middle-income countries (LMICs). | 26,60,62,92,113,115,118,127,128,134,173,204,207,228 |
| 11 | **Partner with patient advocacy organizations**  Incorporate perspectives of pregnant women into the design, implementation, and analysis of drug and vaccine trials. Their expertise can optimise study design, enhance recruitment and retention, and improve data collection accuracy and completeness. | 7,68,71 |
| 12 | **Establish dedicated models for research, capacity strengthening, and funding**  Prioritise dedicated funding models for drug and vaccine development for pregnant and lactating populations, covering all stages from preclinical research through to post marketing safety monitoring. These funding models should include streamlined legal and regulatory pathways, support for both investigator-and pharmaceutical company-initiated drug development, incentives such as extension of patent privileges, and strategies to encourage drug uptake like adequate insurance reimbursement. Professional bodies should develop research agendas that advance drug development in this area. In LMICs, investments should be directed towards establishing centers of excellence to foster expertise, enhance infrastructure, and improve data collection. Establish global and regional research networks to leverage shared resources, expertise and data. Expand the clinical trial workforce with specialists in maternal-fetal medicine, obstetrics, and lactation pharmacology. Train key stakeholders on conducting clinical trials in pregnancy and lactation, emphasising ethical and legal considerations. | 29,60,62,89,92,98,99,107,128,134,136,204 |
| 13 | **Incorporate strategies for liability mitigation**  Risk-sharing strategies and liability mitigation mechanisms can incentivise greater involvement of pharma companies in drug development for pregnant and lactating populations. Establish compensation plans for research-related injuries to motivate investments and participation in clinical trials. | 89,90,103,128 |
| 14 | **Enhance ethics committee capacity and decision-making**  Index ethics publications with relevant keywords for better accessibility. Provide ethics committees with clear guidelines to promote responsible inclusion with clear justifications for inclusion and exclusion criteria. Ethics committees must establish evidentiary standards and conduct reviews to support decision-making that promotes participant safety. Ethics committees should consult with maternal-fetal medicine specialists when assessing minimal risk, and professional bodies should define what constitutes 'minimal risk' to aid this evaluation. Share case studies and research insights for practical solutions to ethical challenges. Provide clear guidance on balancing risk-benefits, consent of the non-birthing parent, and respecting reproductive choice. Include diverse members on ethics committees, including representatives of participant groups, and solicit community input. | 38,42,60,74,92,99,115,124,207,218 |
| 15 | **Collaborate to support data sharing**  Strengthen networks of individuals and organisations advocating for the inclusion of pregnant and lactating women in clinical research. Develop practical guidance on ways to apply global consensus on the topic, with flexibility for making context-specific adjustments. Use guidance from the Global Alignment of Immunization and Antiviral Guidance (GAIA) to prioritise relevant data collection across diverse settings, including in LMICs. | 60,150 |
